# Myocardial infarction and individual nonsteroidal anti-inflammatory drugs meta-analysis of observational studies

**DOI:** 10.1002/pds.3437

**Published:** 2013-04-25

**Authors:** Cristina Varas-Lorenzo, Nuria Riera-Guardia, Brian Calingaert, Jordi Castellsague, Francesco Salvo, Federica Nicotra, Miriam Sturkenboom, Susana Perez-Gutthann

**Affiliations:** 1 RTI Health SolutionsBarcelona, Spain; 2 RTI Health SolutionsResearch Triangle Park, NC, United States; 3 Universite Victor SegalenBordeaux, France; 4 Department of Statistics, Biostatistics and Epidemiology Unit, University of Milano-BicoccaMilan, Italy; 5 Erasmus University Medical CenterRotterdam, Netherlands

**Keywords:** myocardial infarction, meta-analysis, observational studies, anti-inflammatory agents, non-steroidal, epidemiology, pharmacoepidemiology

## Abstract

**Objective:**

To conduct a systematic review of observational studies on the risk of acute myocardial infarction (AMI) with use of individual nonsteroidal anti-inflammatory drugs (NSAIDs).

**Methods:**

A search of Medline (PubMed) for observational studies published from 1990 to 2011 identified 3829 articles; 31 reported relative risk (RR) of AMI with use of individual NSAIDs versus nonuse of NSAIDs. Information abstracted in a standardized form from 25 publications was used for the meta-analysis on 18 independent study populations.

**Results:**

Random-effects RR (95% confidence interval (CI)) was lowest for naproxen 1.06 (0.94–1.20), followed by celecoxib 1.12 (1.00–1.24), ibuprofen 1.14 (0.98–1.31), meloxicam 1.25 (1.04–1.49), rofecoxib 1.34 (1.22–1.48), diclofenac 1.38 (1.26–1.52), indometacin 1.40 (1.21–1.62), etodolac 1.55 (1.16–2.06), and etoricoxib 1.97 (1.35–2.89). Heterogeneity between studies was present. For new users, RRs (95% CIs) were for naproxen, 0.85 (0.73–1.00); ibuprofen, 1.20 (0.97–1.48); celecoxib, 1.23 (1.00–1.52); diclofenac, 1.41 (1.08–1.86); and rofecoxib, 1.43 (1.21–1.66).

Except for naproxen, higher risk was generally associated with higher doses, as defined in each study, overall and in patients with prior coronary heart disease. Low and high doses of diclofenac and rofecoxib were associated with high risk of AMI, with dose–response relationship for rofecoxib. In patients with prior coronary heart disease, except for naproxen, duration of use ≤3 months was associated with an increased risk of AMI.

**Conclusions:**

Most frequently NSAIDs used in clinical practice, except naproxen, are associated with an increased risk of AMI at high doses or in persons with diagnosed coronary heart disease. For diclofenac and rofecoxib, the risk was increased at low and high doses. Copyright © 2013 John Wiley & Sons, Ltd.

## INTRODUCTION

The cardiovascular safety of nonsteroidal anti-inflammatory drugs (NSAIDs) is still under scrutiny after the introduction of selective cyclooxygenase-2 (COX-2) inhibitors.^1^–^3^ The United States (US) Food and Drug Administration and the European Medicines Agency reviewed the safety of selective COX-2 inhibitors, resulting in their contraindication in patients with ischemic heart disease, stroke, or peripheral arterial disease.

Syntheses of published interventional and observational studies conclude that both selective and nonselective COX-2 inhibitors increase the risk of acute myocardial infarction (AMI), and this risk varies across individual NSAIDs.^4^–^7^ Cardiovascular toxicity associated with selective COX-2 and some traditional NSAIDs is mediated through a common mechanism involving the inhibition of COX-2-dependent prostacyclin. Naproxen, at high doses in some individuals, is the only nonaspirin NSAID that lacks functional COX-2 selectivity in platelets.^8^,^9^

Within the Safety of NSAID (SOS) project, a research and development project funded by the Directorate General of Research and Innovation of the European Commission under the Seventh Framework Programme, we performed a quantitative systematic literature review of observational studies assessing the risk of cardiovascular events associated with the use of NSAIDs. (http://www.sos-nsaids-project.org).

## METHODS

### Data sources, data extraction, and quality assessment

We performed a systematic literature search on cardiovascular events in the Medline database (PubMed) using free-text search terms and Medical Subject Headings for myocardial infarction, acute coronary syndrome, sudden cardiac death, stroke, heart failure, left ventricular dysfunction, and nonsteroidal anti-inflammatory agents (see online material). We examined references of articles for additional sources.

Eligible studies for review were observational cohort or case–control studies published in peer-reviewed journals from January 1, 1990, through May 4, 2011. Our search period started in 1990 because the first epidemiology study on the risk of AMI and NSAIDs was published in 2000.^4^,^6^

We used the Newcastle-Ottawa Scale^10^ to evaluate the selection and comparability of study groups and ascertainment of the exposure in case–control studies or of the outcome in cohort studies. Two investigators (NR and CV) evaluated the quality and methodological limitations of each study for the assessment of potential biases, and discordances were solved by consensus.

### Data synthesis and analysis

Using a standardized form, we extracted the odds ratio (OR) or relative risk (RR) for each individual NSAID estimated in each study from the model that was adjusted for the largest number of factors. The main analysis was conducted on all subjects exposed to individual NSAIDs and all types of AMI events. A dose–response analysis used the reported low-medium and high-dose estimates from each study. Data were limited for the evaluation of the effect of duration for individual NSAIDs. We performed several sensitivity analyses.

We estimated pooled RRs and 95% CI for the effect of each NSAID with at least three point estimates from independent studies, using the inverse variance weighting method.^11^ Fixed and random effects were estimated, but Forest plots were based on the random-effects models. Heterogeneity between studies was assessed by Cochran's *χ^2^* test of homogeneity. Tau^2^ was used to quantify the between-study variance for random-effects models. The Higgins *I^2^* statistic was used to describe the percentage of between-study variability in effect estimates attributable to true heterogeneity rather than chance. The *χ^2^* test was used to test for homogeneity between subgroups. Publication bias was examined by review of funnel plots. The analysis was conducted using Review Manager soft ware (version 5.0.22, The Nordic Cochrane Centre, Copenhagen).

## RESULTS

### Study selection and characteristics of included studies

**Figure 1:**
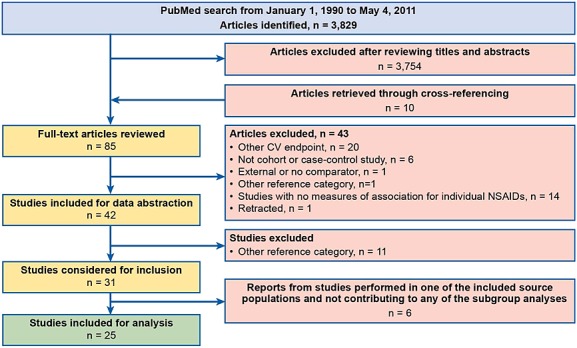
Flow chart of identification and selection of studies. Note: the individual NSAIDs used as reference in each of the 11 excluded studies were the following: diclofenac (n = 2); ibuprofen or diclofenac (n = 1); meloxicam (n = 1); rofecoxib (n = 1); celecoxib (n = 2); acetaminophen (n = 1); aspirin (n = 1); non-naproxen NSAIDS (n = 1); non-selective NSAIDS (n = 1)

For inclusion in the meta-analysis, studies were required to provide measures of association comparing the risk of AMI between users of individual NSAIDs and nonusers or remote NSAID users. The broad search identified 3829 articles; after initial exclusions, the full text of 85 articles was reviewed (Figure 1). A total of 42 articles met the inclusion criteria for study design, outcome of interest, and study medications; of them, 11 were excluded because they used another reference category than non use or remote use of NSAID, resulting in 31 for inclusion (see Table1). Because 20 out of the 31 articles selected for inclusion reported on the same source populations, for each data source, we included the most recent study results for the main analysis (n = 18),^12^–^29^ additional publications (n = 7) provided data for subgroup analyses,^30^–^36^ and the other six did not provide additional information for the analysis (see online material).

Table 1 describes the 25 articles providing data for either the main meta-analysis of AMI (from 18 independent studies) or subgroup analyses. The studies were cohort^12^–^14^,^19^,^24^,^29^,^35^,^36^ or nested case–control^15^,^17^,^18^,^21^–^23^,^25^–^27^,^30^–^34^ studies using automated health databases and involved a large number of study subjects. Three field case–control studies^16^,^20^,^28^ assessed exposure by interviewing patients and controls. The studied populations ranged from low-medium to high risk according to the prior MI or CHD history of participants (Table 1). Half of the studies described the aspirin use, ranging from less than 3% to about 30% of the studied population. The proportion of fatal events varied across studies. The definition of current use was mostly homogeneous, including use at index date or during 7 or 30 days, or less, before the index date.

**Table 1:** Main characteristics of studies included in the meta-analysis

Source population, study period	Population, N, prior MI/CHD (%)	AMI ascertainment	Current use
**Cohort studies**			
Denmark, 1997–2006^35^	N: 83 675; MI: 100%	Fatal/nonfatal recurrent	Five time periods
Denmark, 1997–2005^24^	N: 1 028 427; MI: 0%	First-ever fatal/nonfatal	At index day
Denmark, 1995–2002^36^	N: 58 432; MI: 100%	Recurrent fatal/nonfatal; included OOH CHD deaths	At index date
Medicare, US, 1999–2003^13^	N: 98 370; MI: 7%	Fatal/nonfatal	At index day
US, Canada and UK,^a^ 1999–2004^29^	N: 48 566; CHD: 100%	Fatal/nonfatal, included OOH CHD deaths	At index day
Veterans Administration, US, 2000–2002^12^	N: 384 322; MI: 0.8–1.2%	Fatal/nonfatal	Last 180 days
Medicaid, TN, US, 1999–2001^14^	N: 453 962; NR	Fatal/nonfatal, included OOH CHD deaths	At index day
Ontario, Canada, 1998–2001^19^	N: 166 964; MI: 5%	Fatal/nonfatal	At index day
**Nested or population-based case–control studies**			
Medicare, US, 1991–1995^30^	N: 22 125; MI: 0%	First-ever fatal/nonfatal	Last 180 days
Kaiser Permanente, US, 1999–2001^15^	N: 1 394 764; MI: < 1%	Fatal/nonfatal included OOH CHD deaths	At index day
Saskatchewan, Canada, 1999–2001^17^	N: 364 658; CHD: 16.5%	Fatal/nonfatal, included OOH CHD deaths	Last 7 days
Quebec, Canada, 1999–2002^31^,^32^	N: 125 000; MI: 0% (31)–6.2% (32)	Fatal/nonfatal	At index day
Quebec, Canada, 1999–2002^18^	N: 113 927, MI: 0%	First-ever fatal/nonfatal	At index day
GPRD, UK, 2000–2004^21^	N: 486 378; CHD: 18.2%	Fatal/nonfatal, included OOH CHD deaths	Last 14 or 7 days
GPRD, UK, 1997–2000^33^,^34^	N: 404 183, CHD: 17%	Fatal/nonfatal, included OOH CHD deaths	Last 30 days
THIN, UK, 2000–2005^22^	N: 716 395; NR	Nonfatal	Last 7 days
QResearch, UK, 2000–2004^23^	N: 95 567; MI: 0%	First-ever fatal/nonfatal, included OOH CHD deaths	Last 90 days
PHARMO, The Netherlands, 2001–2004^26^	N: 485 059, CVD: 2.4%	Fatal/nonfatal	At index day
Finland, 2000–2003^25^	N: 172 258; MI: 0%	First-ever fatal/nonfatal	At index day
Denmark, 2000–2003^27^	N: 113 077; MI: 0%	First-ever fatal/nonfatal	Last 30 days
**Hospital field case–control studies**			
Philadelphia, US, 1998–2002^16^	N: 8518; MI: 0%	First-ever nonfatal	Last 7 days
Newcastle Australia, 2003–2004^20^	N: 806; NR	Fatal/nonfatal ACS	Last 7 days
Spain, 2007^28^	N: 5908; MI: 5%	Fatal/nonfatal ACS	Last 7 days

a Three cohorts: Medicaid TN, US; Saskatchewan, Canada; GPRD, UK. Duration subanalysis of the study from García-Rodríguez 2004. ACS = acute coronary syndrome; AMI = acute myocardial infarction; CHD = coronary heart disease; GPRD = General Practice Research Database; MI = myocardial infarction; NR = not reported; OOH = out of hospital; TN = Tennessee; UK = United Kingdom; US = United States of America;

Note: First-ever AMI denotes the occurrence of the first AMI during the study period among patients without prior history of diagnosed MI. Recurrent AMI denotes the recurrence of an AMI among patients identified at the time of the qualifying AMI. Otherwise, AMI denotes the first occurrence of an AMI during the follow-up period among patients with and without prior history of a diagnosed MI.

### Quality of studies

All except six studies^12^–^14^,^19^,^20^,^26^ reported a good selection and definition of subjects (see online material). One study reported results from an exposed cohort of patients who were dispensed at least two successive NSAID prescriptions for at least 30 days and followed for only 1 year.^19^ Immortal time bias was therefore present in this study. Two of the field case–control studies might have misclassified the exposure;^16^,^20^ in one, exposure was ascertained differently in cases (up to 7 days after the index date) than in controls (up to 4 months after the index date), likely resulting in differential misclassification of exposure.^16^

### Meta-analysis results

The random-effects summary estimate (RR; 95% CI) of the risk of AMI was lowest for naproxen (1.06; 0.94–1.20), followed by celecoxib (1.12; 1.00–1.24) and ibuprofen (1.14; 0.98–1.31). Meloxicam (1.25; 1.04–1.49), rofecoxib (1.34; 1.22–1.48), diclofenac (1.38; 1.26–1.52), indometacin (1.40; 1.21–1.62), etodolac (1.55; 1.16–2.06), and etoricoxib (1.97; 1.35–2.89) were associated with an increased risk of AMI (eFigure 1). Fixed models produced summary estimates of similar magnitude but with more precision than random-effect models. Because heterogeneity was present across studies, we present only estimates under the random-effects models. There were no differences in the subgroup analyses between pooled estimates according study design. Field case–control studies provided very heterogeneous results, and the pooled estimates had wider 95% confidence limits; two of these studies reported very low ORs.^16^,^20^

Table 2 summarizes results of analyses for the most frequently used NSAIDs, overall (all types of AMI) and restricted to first-ever incident cases, new users, or high-risk populations with prior coronary heart disease. First-ever incident AMI denotes the first occurrence of an AMI during the follow-up among patients without prior history of MI. New users were defined in each study by excluding prevalent users from the study analysis or including only cohorts of new users in the study.

**Table 2:** Summary relative risk (random effects) of acute myocardial infarction for frequently used NSAIDs, overall and restricted analyses

All study designs	Summary relative risk (95% CI)				
	
	Naproxen	Ibuprofen	Diclofenac	Celecoxib	Rofecoxib
**AMI**^a^	**1.06**	**1.14**	**1.38**	**1.12**	**1.34**
	**(0.94, 1.20)**	**(0.98, 1.31)**	**(1.26, 1.52)**	**(1.00, 1.24)**	**(1.22, 1.48)**
	(n = 17)	(n = 13)	(n = 11)	(n = 18)	(n = 17)
*Heterogeneity (P value)*	*<0.00001*	*<0.00001*	*0.005*	*<0.0001*	*0.0005*
**First- Ever AMI**^b^	**1.00**	**1.18**	**1.38**	**1.10**	**1.41**
	**(0.79, 1.26)**	**(1.01, 1.38)**	**(1.20, 1.60)**	**(0.90, 1.36)**	**(1.25, 1.59)**
	(n = 7)	(n = 4)	(n = 3)	(n = 6)	(n = 6)
*Heterogeneity (P value)*	*<0.0001*	*0.001*	*0.05*	*0.002*	*0.11*
**New users**^c^	**0.85**	**1.20**	**1.41**	**1.23**	**1.43**
	**(0.73, 1.00)**	**(0.97, 1.48)**	**(1.08, 1.86)**	**(1.00, 1.52)**	**(1.23, 1.66)**
	(n = 7)	(n = 4)	(n = 4)	(n = 8)	(n = 10)
*Heterogeneity (P value)*	*0.31*	*0.006*	*0.01*	*<0.00001*	*0.0009*
**New users, at index date**^d^	**0.82**	**1.15**	**1.71**	**1.06**	**1.33**
	**(0.71, 0.95)**	**(0.94, 1.40)**	**(1.38, 2.12)**	**(0.89, 1.27)**	**(1.11, 1.58)**
	(n = 5)	(n = 3)	(n = 2)	(n = 5)	(n = 6)
*Heterogeneity (P value)*	*0.53*	*0.08*	*0.20*	*0.04*	*0.01*
**High-risk populations**^e^	**1.13**	**1.32**	**1.34**	**1.28**	**1.37**
	**(0.87, 1.46)**	**(1.14, 1.52)**	**(0.91, 1.98)**	**(0.99, 1.64)**	**(1.06, 1.79)**
	(n = 5)	(n = 3)	(n = 4)	(n = 5)	(n = 5)
*Heterogeneity (P value)*	*0.12*	*0.28*	*0.0002*	*0.0003*	*0.003*

Note: Reference group, no or remote NSAID use. Studies included in each analysis:

a AMI;^12^–^29^

b First-ever AMI;^16^,^18^,^21^,^23^,^25^,^27^,^30^,^33^

c New users;^13^,^14^,^17^,^19^,^21^,^22^,^24^,^27^,^29^,^31^

d New users at index date;^13^,^14^,^19^,^24^,^29^,^31^

e High-risk populations (populations with prior diagnosed AMI or CHD).^17^,^21^,^29^,^32^,^33^,^35^ AMI = acute myocardial infarction.

Note: First-ever AMI denotes the occurrence of the first event during the study period among patients without prior history of diagnosed MI. Otherwise, AMI denotes the first occurrence of an AMI during the follow-up period among patients with and without prior history of a diagnosed MI.

The pooled estimates (RR; 95% CI) for new users were estimated for naproxen (0.85; 0.73–1.00), ibuprofen (1.20; 0.97–1.48), diclofenac (1.41; 1.08–1.86), celecoxib (1.23; 1.00–1.52), and rofecoxib (1.43; 1.23–1.66) (Table 2). The summary estimates restricted to studies defining new current users as those who were exposed at the index date provided similar results (Table 2).

Restricting the analysis to studies that included out-of-hospital (community) coronary heart disease deaths in the definition of AMI^14^,^15^,^17^,^21^,^23^,^29^ yielded the following pooled estimates: naproxen, 1.09; ibuprofen, 1.09; celecoxib, 1.09; diclofenac, 1.37; and rofecoxib, 1.26. Information was inadequate to provide a pooled analysis stratified by fatal and nonfatal cases.

Studies covering early periods after the introduction of COX-2 inhibitors (from 1999 to 2002) yielded, for celecoxib, significantly lower summary RR (95% CI) estimates (0.97; 0.82–1.14) than the studies that covered a more extended period, through 2005, (1.21; 1.06–1.51) (*P* = 0.03),

We did not find differences between the summary estimates from studies conducted in the US, Canada, or Europe (data not shown).

#### Dose effect

Overall, 11 studies reported the effect of individual NSAID dose on the risk of AMI.^14^,^15^,^17^,^18^,^21^,^22^,^24^,^26^,^29^,^32^,^36^ Most studies used similar cut-off values to define low-medium and high daily doses, except for naproxen, for which definitions varied widely across studies. Three studies^14^,^17^,^29^ defined doses using slightly higher cut-off values than the other studies for all NSAIDs except rofecoxib (see online material).

**Figure 2:**
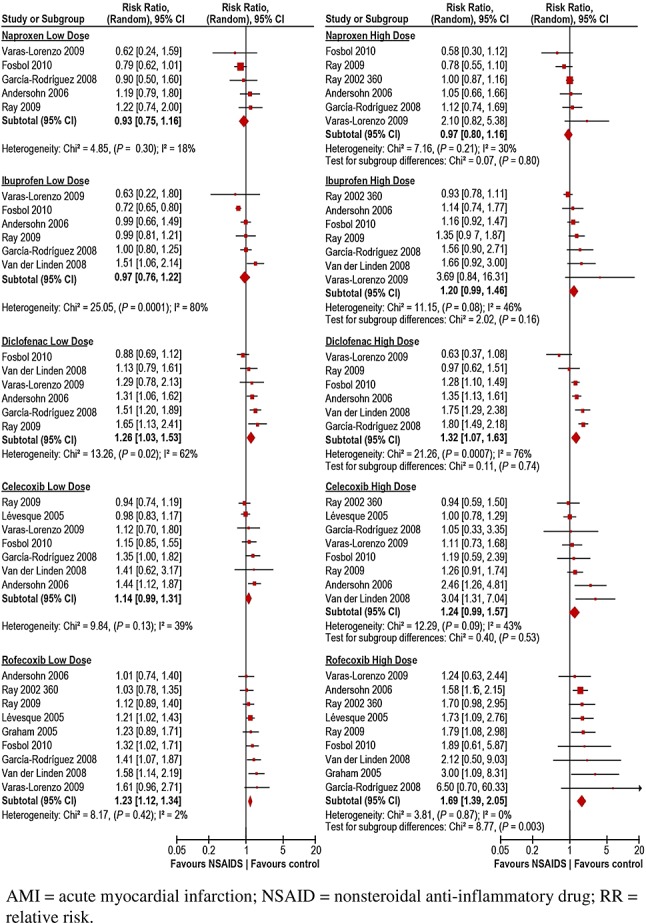
Pooled relative risk (random effects) of acute myocardial infarction associated with current use of individual NSAIDs relative to NSAID nonuse, according to dose group. AMI = acute myocardial infarction; NSAID = nonsteroidal anti-inflammatory drug; RR = relative risk

Forest plots for the risk of AMI by dose for naproxen, ibuprofen, celecoxib, diclofenac, and rofecoxib compared with nonusers are in Figure 2. Except for naproxen, a tendency to higher risk was generally associated with higher doses, as defined in each study. Low and high doses of diclofenac and rofecoxib were associated with higher risk of AMI, but dose–response relationship was present only for rofecoxib (Figure 2). Heterogeneity between studies was reduced in the dose analysis. Similar results were observed in a sensitivity analysis that included only studies using the same cut-off point to define high dose (data not shown).

#### Duration effect

Few studies reported on the effects of treatment duration on the risk of AMI.^17^,^21^,^29^,^31^,^34^,^35^ No consistent pattern was observed across studies. For diclofenac, one study reported the highest RR with long-term duration of use.^34^ For celecoxib, rofecoxib, and etoricoxib, the highest RR were observed with shortest durations. Definitions varied across studies and prevented pooling of the effect estimates (see online material), except in the subgroup analysis restricted to studies performed in high-risk population (see following section).

#### High-risk populations

### Prior CHD history

**Figure 3:**
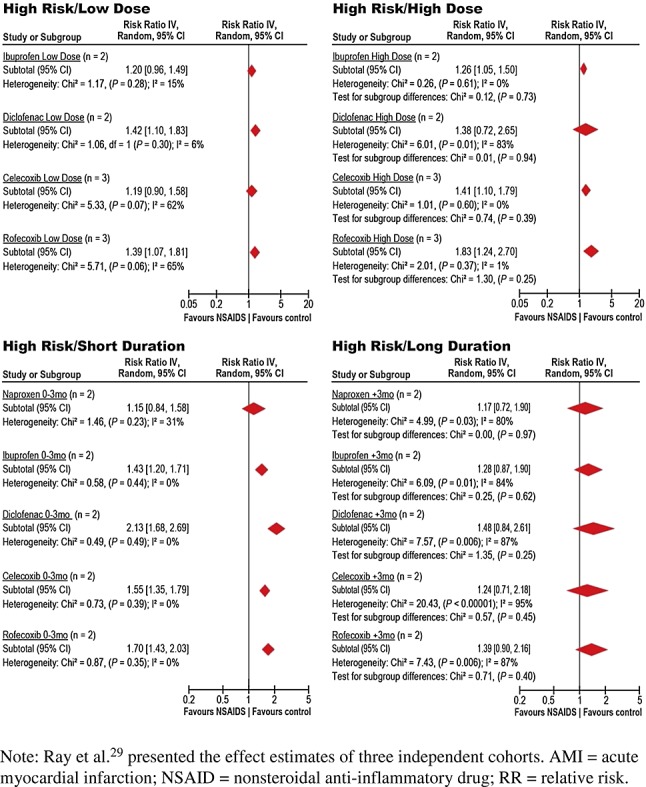
Pooled relative risk (random effects) of acute myocardial infarction associated with current use of individual NSAIDs relative to NSAID nonuse according to dose group and duration, in populations with preexisting diagnosed coronary heart disease. Note: Ray *et al*.^29^ presented the effect estimates of three independent cohorts. AMI = acute myocardial infarction; NSAID = nonsteroidal anti-inflammatory drug; RR = relative risk; Duration in months

Table 2 presents results from six studies evaluating the risk of AMI in high-risk populations by CHD history.^17^,^21^,^29^,^32^,^33^,^35^ The population was stratified by prior history of AMI^32^ or of CHD.^17^,^21^,^33^ One publication^29^ reported the results of three cohorts (US, Canada, and UK) identified immediately after AMI, unstable angina, or coronary revascularization procedure. One study^35^ presented the risk of recurrent AMI and death. The pooled RR (95% CI) were for naproxen (1.13; 0.87–1.46), ibuprofen (1.32; 1.14–1.52), celecoxib (1.28; 0.99–1.64), diclofenac (1.34; 0.91–1.98), and rofecoxib (1.37; 1.06–1.79). Three of these studies^29^,^32^,^36^ provided data on dose effect, and two^29^,^35^ on treatment duration including one study^29^ that presented information from three population-based cohorts. High doses were associated with a high risk of AMI for ibuprofen and celecoxib; for diclofenac and rofecoxib, both low and high doses were associated with a high risk of AMI (Figure 3).

Except for naproxen, the estimates of risk for each of the evaluated individual NSAIDs in the subgroup of shorter duration were associated with an increase of AMI in patients with prior history of coronary heart disease (Figure 3).

### Concomitant aspirin use

Five studies presented analysis results in patients using aspirin concomitantly with the most frequently used individual NSAIDs,^16^,^18^,^22^,^32^,^33^ but two studies presented results from the same source population.^18^,^32^ Pooled RRs (95% CI) for each individual NSAID with or without concomitant use of aspirin were for naproxen, 0.95 (0.61–1.47) and 1.22 (0.78–1.92); celecoxib, 0.90 (0.73–1.10) and 0.94 (0.58–1.55); and rofecoxib, 1.14 (0.93–1.41) and 1.38 (1.17–1.63). Based in fewer than three studies, for ibuprofen, 1.15 (0.88–1.50) and 1.02 (0.79–1.31); meloxicam, 1.02 (0.52–2.00) and 1.23 (0.52–2.94); and finally for diclofenac, 1.29 (1.02–1.63) versus 1.79 (1.51–2.11) (X^2^ = 4.96; p = 0.03).

#### Assessment of potential publication bias—Funnel plots

The funnel plots generated by graphing RR against the standard error of the log of RR appear quite symmetric for all five of the individual NSAIDs (see online material). Relatively few small studies were identified, and all of them found null associations (except for celecoxib in one study). As expected, in a few cases, some of the smaller studies had more extreme RR; this does not necessarily suggest publication bias but instead could reflect that the smaller study was of lesser quality or was perhaps conducted among a particularly high-risk population.^37^ These findings argue against the presence of publication bias.

## DISCUSSION

This meta-analysis of approximately 100 000 AMI events from 18 independent study populations and 64 000 AMI events in the subgroup analyses supports variability in the risk of AMI associated with current use of the most frequently used NSAIDs in comparison with nonuse of NSAIDs. Except for naproxen, almost all NSAIDs most frequently used in clinical practice, if used at high doses or in populations with prior CHD, are associated with an increased risk of AMI. Rofecoxib and diclofenac used at either low or high doses are associated with an elevated risk of AMI, but higher doses of rofecoxib are associated with higher risk of AMI than low doses.

Our results were consistent with those of previous meta-analyses. Meta-analyses of randomized clinical trials reported an increased risk of vascular events associated with selective COX-2 inhibitors, largely attributable to a two-fold increased risk of AMI^5^. High-dose regimens of diclofenac and ibuprofen, but not naproxen, were associated with similar excess risk. The authors acknowledged that the quality of the reported safety data from these trials was suboptimal. Poor quality of reported safety data in clinical trials prevented pooling of results.^38^

Estimated risks of AMI were of similar magnitude and trend to those estimated in prior meta-analyses of observational studies but provide further insights on dose effects and effects in populations with prior coronary heart disease.^4^,^6^ A recent published meta-analysis of observational studies focused on the overall cardiovascular risk associated with NSAIDs, included a variety of individual endpoints such as AMI, stroke, recurrent AMI and all-cause mortality, or the composite of AMI and stroke endpoints.^39^ The large number of included studies and events improved precision of the summary estimates, but studies were very heterogeneous, mostly due to the variety of outcomes and type of included populations.

Our meta-analysis was restricted to acute coronary events occurring in independent populations. A separate publication reported the results of a meta-analysis on the risk of all subtypes of stroke and ischemic stroke associated with the use of individual NSAIDs.^40^ The separate evaluation of the risk of AMI and stroke is important before combining coronary and cerebrovascular outcomes. Reliable interpretation of the combined results requires a relatively small gradient of the magnitude of the effect across disease components (i.e. AMI, stroke, death) and of their clinical relevance for individual patients. To separate subtypes of events with different pathophysiology, such hemorrhagic stroke, that can potentially be associated with differential effects with the same individual medication, is of importance.

**Figure 4:**
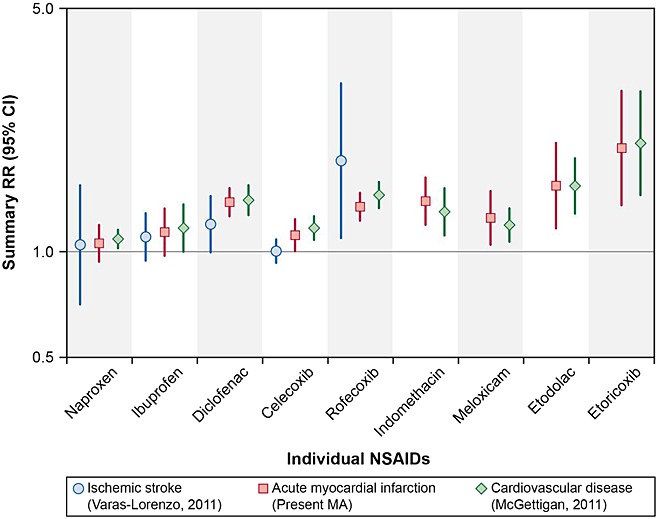
Summary relative risk of acute myocardial infarction, stroke, or combined cardiovascular endpoints for individual NSAIDs compared with NSAID nonuse from three independent meta-analyses. Data sources: acute myocardial infarction (current meta-analysis), stroke,^40^ and combined cardiovascular endpoint^39^

Figure 4 displays the summary RR for ischemic stroke or AMI obtained from our two separate meta-analyses and also for the combined cardiovascular endpoints reported by McGettigan and Henry.^39^ From our meta-analyses, the direction and magnitude of the summary RR suggest a similar but agent-specific thrombotic effect on the coronary and cerebrovascular system. However, the clinical relevance are expected to be different for each individual patient due to prognostic and quality-of-life differences for stroke and AMI.

The extent of COX-2-dependent prostacyclin inhibition may represent an independent key determinant of the increased thrombotic risk with NSAIDs in the presence of insufficient COX-1 activity (< 95%) to inhibit platelet function.^9^ Individual NSAIDs with a degree of COX-2 inhibition less than 90% at therapeutic concentrations (ibuprofen, meloxicam, celecoxib, and etoricoxib) had RR of AMI of 1.18 (95% CI, 1.02–1.38), whereas those with greater COX-2 inhibition (rofecoxib, diclofenac, indometacin, and piroxicam) had RR of 1.60 (95% CI, 1.41–1.81).^21^ A similar result was obtained in the evaluation of the occurrence of first AMI in new users of NSAIDs by COX-2 selectivity.^41^ However, other mechanisms associated with the use of individual NSAIDs, such as effects mediated through the renal system and increases in arterial blood pressure, could be implicated in the variability of the risk of AMI or stroke across individual NSAIDs.

### Limitations: Role of biases in included studies

Most of our analyses detected heterogeneity between effect estimates obtained across studies for all the frequently used NSAIDs. We reported random-effects estimates, as recommended in the context of substantial heterogeneity, and for some individual NSAIDs, Tau-squared was still acceptable.

The main limitations of the present meta-analysis descend from the limitations of each of the included studies. Observational studies are prone to confounding, selection, and information bias.

*Residual confounding* can be a major limitation for the majority of the included studies since the magnitude of the increased risks was rather small. Residual confounding might be present in studies that failed to systematically record some life style factors. Few studies adjusted the analysis for socioeconomic status^19^,^22^,^23^,^36^ or for education and physical activity.^16^,^42^ In a separate survey performed on members of one source population, users of COX-2 inhibitors were more likely than nonusers to have lower educational attainment, obesity, and current smoking^13^; investigators estimated that these differences caused a 2% bias away from the null.

*Confounding by indication* could have operated in opposite directions over the years. The majority of studies accounted for available baseline risk factors for cardiovascular disease. Bias by contraindication to patients with high cardiovascular risk would be minor because only one study included time after the withdrawal of rofecoxib.^28^ In this field study, population size did not allow for precise risk estimates for individual NSAIDs.^28^ The study reported an increased risk of unstable angina and non-ST-segment elevation MI, but not ST-segment elevation MI, associated with overall NSAID use.^28^ The authors hypothesized that NSAID-related thrombosis might be less severe than spontaneous thrombosis. Fatal events occurring before hospital arrival were not included. Survival bias might partially explain the results since, on autopsy, most sudden cardiac death victims in the community had a high-grade coronary stenoses, acute coronary lesions, or prior silent MI.^43^ Heart failure and atrial fibrillation, events that can be triggered by NSAIDs, are associated with an increased probability of dying from CHD outside the hospital.^44^ Lack of ascertainment of such fatalities might have underestimated the risk associated with NSAIDs.

*Protopathic bias* may occur if exposure to the drug of interest started, stopped, or changed because of an unrecognized manifestation of the disease under study. 45 Indication or protopathic bias was present for the risk of AMI associated with the use of NSAIDs in a small subgroup of patients prescribed NSAIDs for unrecognized preinfarction angina.^33^ Protopathic bias can be differential across the individual NSAIDs, especially for those used most frequently for acute pain control.^17^

Inclusion of prevalent NSAID users leads to *survival bias* and by the inability to control for risk factors that may be modified by NSAIDs.^46^,^47^ Analysis of new users supported an increased risk of AMI for the most frequently used individual NSAIDs but not for naproxen.

One study was affected by *immortal time bias* since occurrence of the outcome after the first NSAID prescription was not captured and the unexposed person-time before the start of follow-up of the exposed cohort was not accounted for, which could have underestimated rate ratios comparing the rate of AMI events during current NSAID use with rates during NSAID nonuse.^19^,^48^

Hypertension can be considered a *causal intermediate factor* since it can be an effect of NSAIDs and is an established risk factor for AMI. Thus, adjusting for hypertension or for the use of concurrent antihypertensive medications during follow-up could underestimate RR.^49^ Most studies adjusted for hypertension only at baseline; studies that stratified by hypertension status did not observe effect modification.^13^,^22^ Electronic health databases are limited in assessing changes in blood pressure over time.

Field case–control studies^16^,^20^,^28^ may have been affected by *recall or information bias*, which results from differential misclassification of exposure between cases and controls. In one study, the time window to recall the exposure was different for cases than for controls, which may have underestimated the RR.^16^,^42^,^49^ Interviews conducted by trained research personnel should be conducted without knowledge of either the disease status or the exposure of interest, but this is very difficult to accomplish.^20^ This can result in misclassification bias for either exposure or disease; if different for cases and controls, the misclassification will be differential, and effect estimates may be biased.

*Misclassification of exposure* may be present since information on the use of over-the-counter (OTC) drugs is not recorded in electronic data. A study assessed use of low-dose aspirin and OTC NSAIDs through a standardized telephone survey and did not find differences in the use of OTC drugs between the individual NSAIDs studied.^15^ Based on these published results, although all studies included in the meta-analysis might be affected by misclassification of OTC NSAID use and aspirin use, the bias is likely not differential between individual NSAIDs.

Our subgroup analysis of individual NSAIDs stratified by the concomitant use of aspirin was based on few studies. These very limited results suggested that concomitant use of aspirin might mitigate some, but not all, of the increased risk of AMI associated with diclofenac and likely with rofecoxib. Further evaluation is warranted.

## CONCLUSION

Results from observational studies in this meta-analysis confirm variability of effect of individual NSAIDs on the risk of AMI and that almost all of the NSAIDs most frequently used in clinical practice, except naproxen, are associated with an increased risk of AMI at high doses or when used in persons with diagnosed preexisting coronary heart disease. For diclofenac and rofecoxib, this increased risk is present both at low and high doses.

Ongoing large studies, such as the multi-database observational study within the SOS project, might help to elucidate the risk of AMI associated with individual NSAIDs used in different European populations, by dose and duration. Individual studies within this project were performed with similar study design and definitions to minimize heterogeneity across study populations and databases.

## SUPPORTING INFORMATION

eTable 1. Main characteristics of published studies on the risk of acute myocardial infarction associated with use of individual NSAIDs compared with nonuse or past NSAID use, by geographic area

eTable 2. Study definitions of low and high dose for individual NSAIDs

eTable 3. NSAID duration in studies included in meta-analysis

eTable 4a. Quality assessment according the Newcastle-Ottawa scale: case-control studies

eTable 4b. Quality assessment according the Newcastle-Ottawa scale: cohort studies

eTable 5. MOOSE checklist

eTable 6. Literature search strategy

eTable 7. Excluded studies, by reason for exclusion

eFigure 1. Forest plots for cohort and nested case-control studies, ever had an acute myocardial infarction

eFigure 2. Funnel plot, relative risk of acute myocardial infarction for individual NSAID compared with NSAID nonuse—naproxen

eFigure 3. Funnel plot, relative risk of acute myocardial infarction for individual NSAID compared with NSAID nonuse—ibuprofen

eFigure 4. Funnel plot, relative risk of acute myocardial infarction for individual NSAID compared with NSAID nonuse—meloxicam

eFigure 5. Funnel plot, relative risk of acute myocardial infarction for individual NSAID compared with NSAID nonuse—diclofenac

eFigure 6. Funnel plot, relative risk of acute myocardial infarction for individual NSAID compared with NSAID nonuse—indometacin

eFigure 7. Funnel plot, relative risk of acute myocardial infarction for individual NSAID compared with NSAID nonuse—etodolac

eFigure 8. Funnel plot, relative risk of acute myocardial infarction for individual NSAID compared with NSAID nonuse—celecoxib

eFigure 9. Funnel plot, relative risk of acute myocardial infarction for individual NSAID compared with NSAID nonuse—rofecoxib

eFigure 10. Funnel plot, relative risk of acute myocardial infarction for individual NSAID compared with NSAID nonuse—etoricoxib

eList. References for Supplemental Material
