# Supplementary material for: Myocardial infarction and individual nonsteroidal anti-inflammatory drugs meta-analysis of observational studies
Source: Pharmacoepidemiol Drug Saf. 2013 Apr 25;22(6):559–70. doi: 10.1002/pds.3437 (PMC3749466; doi:10.1002/pds.3437)
Supplement: Supplementary file 1 [file pds0022-0559-SD1.docx]

# Online Supplemental Material

# Myocardial Infarction and Individual Nonsteroidal Anti-inflammatory Drugs: Meta‑analysis of Observational Studies

Varas-Lorenzo C, Riera-Guardia N, Calingaert B, Castellsague J, Salvo F, Nicotra F, Sturkenboom M, Perez-Gutthann S.

## List of Tables

[eTable 1. Main characteristics of published studies on the risk of acute myocardial infarction associated with use of individual NSAIDs compared with nonuse or past NSAID use, by geographic area 3](#_Toc334175639)

[eTable 2. Study definitions of low and high dose for individual NSAIDs 8](#_Toc334175640)

[eTable 3. NSAID duration in studies included in meta-analysis 9](#_Toc334175641)

[eTable 4a. Quality assessment according the Newcastle-Ottawa scale: case-control studies 10](#_Toc334175642)

[eTable 4b. Quality assessment according the Newcastle-Ottawa scale: cohort studies 11](#_Toc334175643)

[eTable 5. MOOSE checklist 12](#_Toc334175644)

[eTable 6. Literature search strategy 16](#_Toc334175645)

[eTable 7. Excluded studies, by reason for exclusion 17](#_Toc334175646)

## List of Figures

[eFigure 1. Forest plots for cohort and nested case-control studies, ever had an acute myocardial infarction 19](#_Toc332890959)

[eFigure 2. Funnel plot, relative risk of acute myocardial infarction for individual NSAID compared with NSAID nonuse—naproxen 22](#_Toc332890960)

[eFigure 3. Funnel plot, relative risk of acute myocardial infarction for individual NSAID compared with NSAID nonuse—ibuprofen 23](#_Toc332890961)

[eFigure 4. Funnel plot, relative risk of acute myocardial infarction for individual NSAID compared with NSAID nonuse—meloxicam 24](#_Toc332890962)

[eFigure 5. Funnel plot, relative risk of acute myocardial infarction for individual NSAID compared with NSAID nonuse—diclofenac 25](#_Toc332890963)

[eFigure 6. Funnel plot, relative risk of acute myocardial infarction for individual NSAID compared with NSAID nonuse—indometacin 26](#_Toc332890964)

[eFigure 7. Funnel plot, relative risk of acute myocardial infarction for individual NSAID compared with NSAID nonuse—etodolac 27](#_Toc332890965)

[eFigure 8. Funnel plot, relative risk of acute myocardial infarction for individual NSAID compared with NSAID nonuse—celecoxib 28](#_Toc332890966)

[eFigure 9. Funnel plot, relative risk of acute myocardial infarction for individual NSAID compared with NSAID nonuse—rofecoxib 29](#_Toc332890967)

[eFigure 10. Funnel plot, relative risk of acute myocardial infarction for individual NSAID compared with NSAID nonuse—etoricoxib 30](#_Toc332890968)

[References for Supplemental Material 31](#_Toc326677142)

eTable 1. Main characteristics of published studies on the risk of acute myocardial infarction associated with use of individual NSAIDs compared with nonuse or past NSAID use, by geographic area

| Author, Year | | Source Popula­tion  Study Period | | Study Popula­tion N, Sex, Age (Years) | Study Design | Type of End­point | Endpoint | Endpoint Definition | Case Validation | Dose  Duration  Aspirin | Exposure Assess­ment | Exposure Definition | Reference Category |
| --- | --- | --- | --- | --- | --- | --- | --- | --- | --- | --- | --- | --- | --- |
| United States of America | | | |  |  |  |  |  |  |  |  |  |  |
| Abraham  2007([1](#_ENREF_1))^, a^ | Veterans Administra­tion, US  2000-2002 | | | N: 384,322  Men  65-99 | Cohort | Ever | Hospitalization for acute myocardial infarction (AMI) | Fatal and nonfatal  No inclusion of out-of-hospital (OOH) coronary heart disease (CHD) deaths | External, PPV 92%-96% | No  No  No | Ever use  Electronic prescriptions | Current, use last 180 days | Nonuse of NSAIDs |
| Solomon  2002([2](#_ENREF_2))^, a^ | Medicare, US  1991-1995 | | | N: 22,125  All  No age limits | Nested case-control | Incident | Hospitalization for AMI | Fatal and nonfatal  No inclusion of OOH CHD deaths | External, PPV 97% | No  No  No | Ever use  Filled prescriptions | Current, use last 180 days | Nonuse of NSAIDs |
| Solomon  2004([3](#_ENREF_3)) | Medicare, US  1998-2000 | | | N: 251,298  All  65+ | Nested case-control | Ever | Hospitalization for AMI | Fatal and nonfatal  No inclusion of OOH CHD deaths | Internal, PPV 93% | Yes ^b^  Yes ^b^  No | Ever use  Filled prescriptions | Current, use at index day | No current use |
| Solomon  2006([4](#_ENREF_4))^, a^ | Medicare, US  1999-2003 | | | N: 98,370  All  No age limits | Cohort | Ever | Hospitalization for AMI | Fatal and nonfatal  No inclusion of OOH CHD deaths | External. PPV 94% | No  No  No | New use  Filled prescriptions | Current, use at index day | Nonuse of NSAIDs |
| Ray  2002([5](#_ENREF_5)) | Medicaid, TN, US  1987-1998 | | | N: 262,644  All  50-84 | Cohort | Ever | Hospitalization for AMI | Fatal and nonfatal  Included OOH CHD deaths | External. PPV 90%-95% | Yes  Yes  No | New use  Filled prescriptions | Current, use at index day | Nonuse of NSAIDs |
| Ray  2002([6](#_ENREF_6))^, a^ | Medicaid, TN, US  1999-2001 | | | N: 453,962  All  50-84 | Cohort | Ever | Hospitalization for AMI | Fatal and nonfatal  Included OOH CHD deaths | External. PPV 90%-95% | Yes  No  No | Ever use and new use  Filled prescription | Current, use at index day | Nonuse of NSAIDs |
| Graham  2005([7](#_ENREF_7))^, a^ | Kaiser Perma­nente, US  1999-2001 | | | N: 1,394,764  All  18-84 | Nested case-control | Ever | Hospitalization for AMI | Fatal and nonfatal  Included OOH CHD deaths | External, PPV 92%-95% | Yes  No  No | Ever use  Filled prescription | Current, use at index day | Past use of NSAIDs |
| Kimmel  2004([8](#_ENREF_8)) | 36 hospitals, Philadel­phia, US  1998-2001 | | | N: 5,208  All  40-75 | Field, hospital case-control | Incident | First hospitalization for AMI | Nonfatal.  No inclusion of OOH CHD deaths | Partial validation in 50%, confirmed 86% | No  No  No | Ever use, self-reported use by question­naires | Current, use last 7 days | Nonuse of NSAIDs |
| Kimmel  2005([9](#_ENREF_9))^, a^ | 36 hospitals, Philadel­phia, US  1998-2002 | | | N: 8,518  All  40-75 | Field, hospital case-control | Incident | First hospitalization for AMI | Nonfatal.  No inclusion of OOH CHD deaths | Partial validation in 50%, confirmed 86% | No  No  Yes | Ever use, self-reported use by question­naires | Current, use last 7 days | Nonuse of NSAIDs |
| Canada |  | | |  |  |  |  |  |  |  |  |  |  |
| Varas-Lorenzo  2009([10](#_ENREF_10))^, a^ | Saskatche­wan Health, Canada  1999-2001 | | | N: 364,658  All  40-84 | Nested case-control | Ever | Hospitalization for AMI | Fatal and nonfatal  Included OOH CHD deaths | Internal, PPV for AMI was 95% for code 410 and 9% for code 411 | Yes  Yes  No | Ever use and new use  Electronic prescriptions | Current, use last 7 days | Nonuse of NSAIDs |
| Lévesque  2005([11](#_ENREF_11))^, a^ | Quebec, Canada  1999-2002 | | | N: 113,927  All  66+ | Nested case-control | Incident | First hospitalization for AMI | Fatal and nonfatal  No inclusion of OOH CHD deaths | External | Yes  No  Yes | Ever use  Filled prescription | Current, use at index day | Nonuse of NSAIDs |
| Lévesque  2006([12](#_ENREF_12))^, a^ | Quebec, Canada  1999-2002 | | | 125,000  All  66+ | Nested case-control | Incident | First hospitalization for AMI | Fatal and nonfatal  No inclusion of OOH CHD deaths | External | No  Yes  No | New use  Filled prescription | Current, use at index day | Nonuse of NSAIDs |
| Brophy  2007([13](#_ENREF_13)) | Quebec, Canada  1999-2002 | | | N: 125,000  All  66+ | Nested case-control | Ever | Hospitalization for AMI | Fatal and nonfatal  No inclusion of OOH CHD deaths | External | Yes  No  Yes | New use  Filled prescription | Current, use at index day | Nonuse of NSAIDs |
| Mamdani  2003([14](#_ENREF_14))^, a^ | Ontario, Canada  1998-2001 | | | N: 166,964  All  66+ | Cohort | Ever | Hospitalization for AMI | Fatal and nonfatal  No inclusion of OOH CHD deaths | External, PPV 89% | No  No  No | New use  Filled prescription | Current, use at index day | Nonuse of NSAIDs |
| Australia |  | | |  |  |  |  |  |  |  |  |  |  |
| McGettigan  2006([15](#_ENREF_15))^, a^ | 2 hospitals in Newcastle, Australia  2003-2004 | | | N: 806  All  No age limits | Field, hospital case-control | Ever | Hospitalization for ACS (AMI or unstable angina) | Fatal and nonfatal  No inclusion of OOH CHD deaths | Internal, through attendant physician | Yes  No  No | Self-reported use by question­naires | Current, use last 7 days | Nonuse of NSAIDs |
| Europe, UK | | |  |  |  |  |  |  |  |  |  |  |  |
| Watson  2002([16](#_ENREF_16)) | | GPRD  UK  1988-1999 | | N: 16,937  All  40-79 | Cohort | Incident | First ever AMI | Fatal and nonfatal  Included OOH CHD deaths | Internal, partial of a 10% sample. Confirmation in 79% | No  No  No | Ever use  Electronic prescriptions | Current, use last 30 days | Nonuse of NSAIDs |
| Schlienger  2002([17](#_ENREF_17)) | | GPRD  UK  1992-1997 | | N: 16,458  All  < 75 | Nested case-control | Incident | First ever AMI | Fatal and nonfatal  Included OOH CHD deaths | External, > 90% confirmation | No  No  No | Ever use  Electronic prescriptions | Current, use at index day | Nonuse of NSAIDs |
| Fischer  2005([18](#_ENREF_18)) | | GPRD  UK  1995-2001 | | N: 42,611  All  < 90 | Nested case-control | Ever | Diagnosis of AMI | Fatal and nonfatal  No inclusion of OOH CHD deaths | External, > 90% confirmed | Yes  Yes  Yes | Ever use  Electronic prescriptions | Current, use at index day | Nonuse of NSAIDs |
| García Rodríguez 2004([19](#_ENREF_19)) | | GPRD  UK  1997-2000 | | N: 404,183  All  50-84 | Nested case-control | Ever (incident-no CHD) | Hospitalization for AMI | Fatal and nonfatal  Included OOH CHD deaths | Internal, partial in a sample, 96% confirmed | No  No  Yes | Ever use  Electronic prescriptions | Current, use last 30 days | Nonuse of NSAIDs |
| García-Rodríguez 2005([20](#_ENREF_20)) | | GPRD  UK  1997-2000 | | N: 404,183  All  50-84 | Nested case-control | Ever | Hospitalization for AMI | Fatal and nonfatal  Included OOH CHD deaths | Internal, partial in a sample, 96% confirmed | No  Yes  No | Ever use  Electronic prescriptions | Current, use last 30 days | Nonuse of NSAIDs |
| Andersohn  2006([21](#_ENREF_21))^, a^ | | GPRD  UK  2000-2004 | | 486,378  All,  40+ | Nested case-control | Ever (incident-no CHD) | First recorded diagnosis of AMI | Fatal and nonfatal  Included OOH CHD deaths | External, PPV > 90%  Review of electronic PP blinded to exposure status | Yes  Yes  No | Ever use and new use  Electronic prescriptions | Current, use last 14 days or 7 days | Nonuse of NSAIDs |
| García-Rodríguez 2008([22](#_ENREF_22))^, a^ | | THIN  UK  2000-2005 | | N: 716,395  All  50-84 | Nested case-control | Ever | Hospitalization for AMI | Nonfatal | Internal, partial in a sample, 95% confirmed | Yes  No  Yes | Ever use and new use  Electronic prescriptions | Current, use last 7 days | Nonuse of NSAIDs |
| Hippisley-Cox  2005([23](#_ENREF_23))^, a^ | | QRe­search  UK  2000-2004 | | N: 95,567  All  25-100 | Nested case-control | Incident | First ever AMI | Fatal and nonfatal  Included OOH CHD deaths | External, 90% confirmation | No  No  No | Ever use  Electronic prescriptions | Current, use last 90 days | Nonuse of NSAIDs |
| Europe, other countries | | | |  |  |  |  |  |  |  |  |  |  |
| Fosbøl  2010([24](#_ENREF_24))^, a^ | | Denmark  1997-2005 | | N: 1,028,437  All  ≥ 10 years | Cohort | Incident | Hospitalization for AMI | Fatal and nonfatal  No inclusion of OOH CHD deaths | External, PPV 93% | Yes  No  No | Ever use  Electronic prescriptions | Current, use at index day | Nonuse of NSAIDs |
| Schjerning Olsen  2011([25](#_ENREF_25))^, a^ | | Denmark  1997-2006 | | N: 83,675  All  30+ | Cohort | Ever | Recurrent MI or death | Fatal and nonfatal  Included OOH CHD deaths | External, PPV 93% | No  Yes  No | Ever and new use  Filled prescription | 5 exposure periods, from index date | Nonuse of NSAIDs |
| Gislason  2006([26](#_ENREF_26)) | | Denmark  1995-2002 | | N: 58,432  All  30+ | Cohort and case-cross­over | Recur­rent | Recurrent MI or death | Fatal and nonfatal  Included OOH CHD deaths | External, PPV > 90% | Yes  No  No | Ever use  Filled prescription | Current, use at index date or last 30 days | Nonuse of specific NSAID |
| Helin-Salmivaara  2006([27](#_ENREF_27))^, a^ | | Finland  2000-2003 | | N: 172,258  All  No age limits | Popula­tion-based case-control | Incident | Hospitalization for AMI | Fatal and nonfatal  No inclusion of OOH CHD deaths | External | No  No  No | Ever use  Filled prescription | Current, use at index day | Nonuse of NSAIDs |
| Van der Linden  2008([28](#_ENREF_28))^, a^ | | PHARMO  The Nether­lands  2001-2004 | | N: 485,059  All  No age limits | Nested case-control | Ever | Hospitalization for AMI | Fatal and nonfatal  No inclusion of OOH CHD deaths | Internal | Yes  No  No | Ever use  Filled prescription | Current, use at index day | Past use of NSAIDs |
| Johnsen  2005([29](#_ENREF_29))^, a^ | | Denmark  2000-2003 | | N: 113,077  All  20+ | Popula­tion-based case-control | Incident | First hospitalization for AMI | Fatal and nonfatal  No inclusion of OOH CHD deaths | External, PPV 90% | No  Yes  No | Ever and new use  Filled prescription | Current, use last 30 days | Nonuse of NSAIDs |
| Bueno  2010([30](#_ENREF_30))^, a^ | | Spain  2007 | | N: 5,908  All  40-85 | Field, hospital case-control | Ever | Hospitalization for ACS (AMI or unstable angina) | Fatal and nonfatal  No inclusion of OOH CHD deaths | Internal | No  No  No | Ever use  Structured question­naire by trained, blinded staff | Current, use last 7 days | Nonuse of NSAIDs |
| Multiple countries | | | |  |  |  |  |  |  |  |  |  |  |
| Ray  2009([31](#_ENREF_31))^, a^ | Tennessee Medicaid, Saskatche­wan Canada, GPRD, UK  1999-2004 | | | N: 48,566  All  40-89 | Cohort | Ever | Hospitalization for AMI | Fatal and nonfatal  Included OOH CHD deaths | External, 92%-95% Tennessee and Saskatche­wan, internal 96% GPRD | Yes  No  No | Ever and new use  Electronic prescriptions | Current, use at index day | Nonuse of NSAIDs |

Abbreviations: ACS, acute coronary syndrome; AMI, acute myocardial infarction; CHD, coronary heart disease; GPRD, General Practice Research Database; MI, myocardial infarction; NSAID, nonsteroidal anti-inflammatory drug; OOH, out of hospital; PP, patient profile; PPV, positive predictive value; THIN, The Health Improvement Network (HE); TN, Tennessee; UK, United Kingdom; US, United States.

^a^ Studies included in the meta-analysis for the overall pooled analysis or subgroup analyses.

^b^ Celecoxib was the reference category for the dose and duration.

eTable 2. Study definitions of low and high dose for individual NSAIDs

|  | Individual NSAID | | | | | | | | | |
| --- | --- | --- | --- | --- | --- | --- | --- | --- | --- | --- |
|  | Naproxen | | Ibuprofen | | Diclofenac | | Celecoxib | | Rofecoxib | |
| Study First Author, Year | Low Dose | High Dose | Low Dose | High Dose | Low Dose | High Dose | Low Dose | High Dose | Low Dose | High Dose |
| Fosbøl, 2010([24](#_ENREF_24)) | ≤ 500 | > 500 | ≤ 1200 | > 1200 | < 100 | ≥ 100 | ≤ 200 | > 200 | ≤ 25 | > 25 |
| Ray, 2009([31](#_ENREF_31)) | < 1000 | ≥ 1000 | ≤ 1600 | > 1600 | < 150 | ≥ 150 | ≤ 200 | > 200 | ≤ 25 | > 25 |
| Van der Linden, 2009([28](#_ENREF_28)) | – | – | ≤ 1200 | > 1200 | ≤ 100 | > 100 | ≤ 200 | > 200 | ≤ 25 | > 25 |
| Varas-Lorenzo 2009([10](#_ENREF_10)) | ≤ 1000 | > 1000 | ≤ 1800 | > 1800 | ≤ 100 | > 100 | ≤ 200 | > 200 | ≤ 25 | > 25 |
| García Rodríguez, 2008([22](#_ENREF_22)) | ≤ 750 | > 750 | ≤ 1200 | > 1200 | ≤ 100 | > 100 | ≤ 200 | > 200 | ≤ 25 | > 25 |
| Brophy, 2007([13](#_ENREF_13)) | – | – | – | – | – | – | ≤ 200 | > 200 | ≤ 25 | > 25 |
| Andersohn, 2006([21](#_ENREF_21)) | ≤ 750 | > 750 | ≤ 1200 | > 1200 | ≤ 100 | > 100 | ≤ 200 | > 200 | < 25 | ≥ 25 |
| Gislason, 2006([26](#_ENREF_26)) | – | – | ≤ 1200 | > 1200 | < 100 | ≥ 100 | ≤ 200 | > 200 | < 25 | ≥ 25 |
| Graham, 2005([7](#_ENREF_7)) | – | – | – | – | – | – | – | – | ≤ 25 | > 25 |
| Lévesque, 2005([11](#_ENREF_11)) | – | – | – | – | – | – | ≤ 200 | > 200 | ≤ 25 | > 25 |
| Ray, 2002([6](#_ENREF_6)) | – | ≥ 1000 | – | ≥ 1800 | – | – | – | ≥ 300 | ≤ 25 | > 25 |

Abbreviation: NSAID, nonsteroidal anti-inflammatory drug.

eTable 3. NSAID duration in studies included in meta-analysis

| Study First Author, Year | NSAIDs Studied | Duration of NSAID Exposure in Studies |
| --- | --- | --- |
| **Olsen, 2011(**[**25**](#_ENREF_25)**)** | naproxen, ibuprofen, diclofenac, celecoxib, rofecoxib | 0-7 days, 7-14 days, 14-30 days, 30-90 days, > 90 days |
| **Ray, 2009(**[**31**](#_ENREF_31)**)** | naproxen, ibuprofen, diclofenac, celecoxib, rofecoxib | < 3 months, 3-12 months, > 12 months |
| **Varas-Lorenzo, 2009(**[**10**](#_ENREF_10)**)** | naproxen, ibuprofen, diclofenac, celecoxib, rofecoxib | 0-30 days, > 30 days |
| **Andersohn, 2006(**[**21**](#_ENREF_21)**)** | naproxen, ibuprofen, diclofenac, celecoxib, rofecoxib | < 3 months, 3-12 months, > 12 months |
| **Lévesque, 2006(**[**12**](#_ENREF_12)**)** | celecoxib, rofecoxib | < 7.8 days, 7.8-29.3 days, 29.4 days-65.7 days, > 65.7 days |
| **García Rodríguez, 2005(**[**20**](#_ENREF_20)**)** | naproxen, ibuprofen, diclofenac, | 0-30 days, 31-365 days, > 365 days |

Abbreviation: NSAID, nonsteroidal anti-inflammatory drug.

eTable 4a. Quality assessment according the Newcastle-Ottawa scale: case-control studies

Among selected studies in the systematic review of AMI risk and individual NSAID use, there were 14 nested or population based case-control studies and 3 field case-control studies for inclusion in the meta-analysis. The results of the quality evaluation according to the Newcastle-Otawa Scale of these studies are presented in the table below for the selection, comparability, and exposure dimensions. Fourteen studies scored the maximum numbers of starts for selection, comparability and exposure.

|  |  | Newcastle-Ottawa Scale Score | | |
| --- | --- | --- | --- | --- |
| Study’s First Author | Publication Year | Selection (max. = 4) | Comparability (max. = 2) | Exposure (max. = 3) |
| Solomon([2](#_ENREF_2)) | 2002 | **** | ** | *** |
| García Rodríguez([19](#_ENREF_19)) | 2004 | **** | ** | *** |
| Garcia Rodriguez([20](#_ENREF_20)) | 2005 | **** | ** | *** |
| Graham([7](#_ENREF_7)) | 2005 | **** | ** | *** |
| Hippisley-Cox([23](#_ENREF_23)) | 2005 | **** | ** | *** |
| Johnsen([29](#_ENREF_29)) | 2005 | **** | ** | *** |
| Kimmel([9](#_ENREF_9)) | 2005 | **** | ** | * |
| Lévesque([11](#_ENREF_11)) | 2005 | **** | ** | *** |
| Andersohn([21](#_ENREF_21)) | 2006 | **** | ** | *** |
| Helin-Salmivaara([27](#_ENREF_27)) | 2006 | **** | ** | *** |
| Lévesque([12](#_ENREF_12)) | 2006 | **** | ** | *** |
| McGettigan([15](#_ENREF_15)) | 2006 | *** | ** | ** |
| Brophy([13](#_ENREF_13)) | 2007 | **** | ** | *** |
| Garcia Rodriguez([22](#_ENREF_22)) | 2008 | **** | ** | *** |
| Van der Linden([28](#_ENREF_28)) | 2008 | *** | ** | *** |
| Varas-Lorenzo([10](#_ENREF_10)) | 2009 | **** | ** | *** |
| Bueno([30](#_ENREF_30)) | 2010 | **** | ** | *** |

eTable 4b. Quality assessment according the Newcastle-Ottawa scale: cohort studies

A total of 8 cohort studies were selected for inclusion in the meta-analysis and their quality was evaluated. The results of this evaluation are presented in the table below for the selection, comparability, and outcome dimensions. Four studies scored four stars, 3 scored three stars, and 1 scored two stars for selection. For comparability, all eight studies scored the maximum (2 stars). And for outcome, all studies but one scored the maximum of 3 stars.

|  |  | Newcastle-Ottawa Scale Score | | |
| --- | --- | --- | --- | --- |
| Study’s First Author | Publication Year | Selection (max. = 4) | Comparability (max. = 2) | Outcome (max. = 3) |
| Ray([6](#_ENREF_6)) | 2002 | *** | ** | *** |
| Mamdani([14](#_ENREF_14)) | 2003 | ** | ** | ** |
| Gislason([26](#_ENREF_26)) | 2006 | **** | ** | *** |
| Solomon([4](#_ENREF_4)) | 2006 | *** | ** | *** |
| Abraham([1](#_ENREF_1)) | 2007 | *** | ** | *** |
| Ray([31](#_ENREF_31)) | 2009 | **** | ** | *** |
| Fosbøl([24](#_ENREF_24)) | 2010 | **** | ** | *** |
| Schjerning Olsen([25](#_ENREF_25)) | 2011 | **** | ** | *** |

eTable 5. MOOSE checklist

| Criteria | | Brief description of how the criteria were handled in the meta-analysis | |
| --- | --- | --- | --- |
| Reporting of background should include | |  | |
| √ | Problem definition | Evidence suggests that nonsteroidal anti-inflammatory drugs (NSAIDs) increase the risk of cardiovascular events. The Safety of Nonsteroidal Anti-inflammatory Drugs (SOS) project is a research and development project funded by the Directorate General of Research and Innovation of the European Commission under the Seventh Framework Programme. Within this project, a quantitative systematic literature review of observational studies assessing the risk of cardiovascular events associated with the use of NSAIDs was performed, with the intention of using the results to plan future research. | |
| √ | Hypothesis statement | The risk of acute coronary syndrome associated with the use of NSAIDs varies across individual NSAIDs. | |
| √ | Description of study outcomes | Acute coronary syndrome, including acute myocardial infarction | |
| √ | Type of exposure or intervention used | Individual NSAIDs compared with nonuse or remote use of NSAID | |
| √ | Type of study designs used | Meta-analysis of observational studies including case-control studies, nested case-control studies, and cohort studies  Studies published only in abstract form, letters and commentaries to the editor, clinical trials, case reports, case series, ecological studies, and studies based on voluntary reporting of events were not considered for inclusion in this systematic review | |
| √ | Study population | Study populations included in individual population-based studies and field hospital studies.  Studies conducted in hospitalized or institutionalized patients were excluded. | |
| Reporting of search strategy should include | | |  |
| √ | Qualifications of searchers | The credentials of the three investigators CVL, JC, and NRG are indicated in the author list. | |
| √ | Search strategy, including time period included in the synthesis and keywords | PubMed from Jan 1990 – May 4, 2011  See Figure 1 in the manuscript and eTable 6 in the appendix online. | |
| √ | Databases and registries searched | Medline and Cochrane | |
| √ | Search software used, name and version, including special features | PubMed used as search engine. EndNote reference management software was used to store retrieved citations and eliminate duplicates | |
| √ | Use of hand searching | Reference lists of relevant articles, including systematic reviews and meta-analyses, were manually searched for additional references. | |
| √ | List of citations located and those excluded, including justifications | Details of the literature search process are outlined in the flow chart (Figure 1 in the manuscript). The complete list of articles reviewed and abstracted is in the appendix eTable 1. The list of excluded studies and reason for exclusion is found in eTable 7. | |
| √ | Method of addressing articles published in languages other than English | Studies published in languages other than English were not included | |
| √ | Method of handling abstracts and unpublished studies | Studies published only in abstract form or unpublished studies were not eligible for inclusion. | |
| √ | Description of any contact with authors | One author was contacted regarding an erroneous confidence interval in a publication. | |
| Reporting of methods should include | |  | |
| √ | Description of relevance or appropriateness of studies assembled for assessing the hypothesis to be tested | Detailed inclusion and exclusion criteria are described in the methods section. | |
| √ | Rationale for the selection and coding of data | Data extracted from each of the studies included population characteristics, study design, exposure, outcome, and possible confounders and effect modifiers of the association. | |
| √ | Documentation of how data were classified and coded | Data were abstracted in standardized form and collected in a Microsoft Access database. | |
| √ | Assessment of confounding | The effect estimate for each individual NSAID, adjusted for the largest set of confounders in each study, was included in the meta-analysis. | |
| √ | Assessment of study quality, including blinding of quality assessors; stratification or regression on possible predictors of study results | The Newcastle-Ottawa Scale (NOS) was used to quantify the methodological quality of each study. In addition, potential methodological issues were evaluated. These were performed by two researchers independently, and discrepancies were resolved by consensus. Analyses by study design, study period, or geographical area were conducted. | |
| √ | Assessment of heterogeneity | Higgins inconsistency *I^2^* statistic was used to describe the percentage of the variability in effect estimates that was due to heterogeneity rather than chance. Tau statistic was also included. Sensitivity and stratified analyses were conducted. Dose effect estimates were more homogeneous across studies. | |
| √ | Description of statistical methods in sufficient detail to be replicated | Description of methods of meta-analyses, sensitivity analyses, and assessment of publication bias are detailed in the methods section. | |
| √ | Provision of appropriate tables and graphics | We included  *In the manuscript:*  Tables: 1. Summary table with characteristics of included studies; 2. Overall results of the main and subgroup analyses  Figures: 1. Flow chart for identification and selection of studies; 2. Forest plot of the analysis by dose; 3. Forest plot (summary estimates) of the analysis by dose and duration in high risk populations; 4. Graph with the summary estimates of 3 independent MA.  *Online supplementary material:*  eTable 1. Summary table of all reviewed studies; eTable 2. Definitions of dose provided in each individual study; eTable 3. NSAID duration categories provided in each individual study; eTable 4a and eTable 4b. Quality of studies evaluated by the NOS; eTable 5 MOOSE checklist; eTable 6. Literature search strategy; e Table 7. Excluded studies and reasons  eFigure 1. Forest plots for the overall analysis; eFigure 2 to 10. Funnel plots for individual NSAIDs | |
| Reporting of results should include | |  | |
| √ | Graph summarizing individual study estimates and overall estimate | eFigure 1; Figure 2 and 3 | |
| √ | Table giving descriptive information for each study included | Table 1 and eTable 1 | |
| √ | Results of sensitivity testing | Table 2, Figure 2 and Figure 3 | |
| √ | Indication of statistical uncertainty of findings | 95% confidence intervals were presented with all summary estimates, p values for homogeneity between subgroups, *I^2^* values and results of sensitivity analyses | |
| Reporting of discussion should include | |  | |
| √ | Quantitative assessment of bias | Assessment of bias is reported in the discussion section. Publication bias was examined by visual evaluation of the funnel plot | |
| √ | Justification for exclusion | Studies conducted in hospitalized or institutionalized patients were excluded because exposure was not well characterized and because of exposure to other comedications that likely differed from that of the overall population.  Published information without a full study report, such as abstracts or letters, was not included because they usually present preliminary results of investigations and data are inadequate for pool analysis. | |
| √ | Assessment of quality of included studies | NOS scores and discussion of methodological limitations of included studies; evaluation of potential reasons for the observed heterogeneity. | |
| Reporting of conclusions should include | |  | |
| √ | Consideration of alternative explanations for observed results | Explanations of the observed results are reported in the discussion section, as well as the degree of consistency with prior systematic reviews of observational studies and clinical trials | |
| √ | Generalization of the conclusions | Only the five most frequently used NSAIDs (naproxen, ibuprofen, diclofenac, celecoxib and rofecoxib) were evaluated in detail. The results indicate the risk of AMI associated with each of these individual NSAIDs as used in clinical practice in North America and the European countries evaluated through the overall time period of observation. | |
| √ | Guidelines for future research | Additional large epidemiology studies to evaluate the risk of AMI associated with most of the individual NSAIDs, by duration, concomitant use of aspirin, and to address the potential for residual confounding, are recommended. | |
| √ | Disclosure of funding source | The research leading to the results of this study has received funding from the European Commission’s Seventh Framework Programme (FP-7) under grant agreement number 223495, the SOS project. The funding source had no role in study design, data collection, data analysis, data interpretation, or writing of the report. | |

eTable 6. Literature search strategy

PubMed was searched using the following groups of search terms, restricted to English-language publications and human subjects.

1. Pertinent, high-level medical subject headings (MeSH) and free-text terms related to cardiovascular events:

- Stroke (hemorrhagic and ischemic), heart failure, myocardial infarction, and associated mortality

2. Pertinent MeSH and free-text terms to identify the relevant drugs.

3. Pertinent MesH and free-text terms related to cohort and case-control studies.

The PubMed Search terms for cardiovascular endpoints are described in the strategy below.

1. stroke [Tiab] OR myocardial infarction [Tiab] OR heart failure [Tiab] OR cardiac failure [Tiab] OR left ventricular dysfunction [Tiab] OR acute coronary syndrome [Tiab] OR cerebrovascular disease [Tiab] OR coronary heart disease [Tiab] OR sudden cardiac death [Tiab] OR cardiovascular mortality [Tiab] OR coronary heart disease [Tiab] OR cardiorenal [Tiab] OR stroke [MeSH] OR myocardial infarction [MeSH] OR heart failure [MeSH] OR ventricular dysfunction, left [MeSH] OR acute coronary syndrome [MeSH] OR death, sudden, cardiac [MeSH]

2. coxibs OR COX2 inhibitors OR cyclooxygenase 2 Inhibitors OR nsaids OR non-steroidal anti-inflammatory OR “Anti-Inflammatory Agents, Non-Steroidal” [Mesh] OR phenylbutazone OR mofebutazone OR oxyphenbutazone OR clofezone OR kebuzone OR indometacin OR sulindac OR tolmetin OR zomepirac OR aceclofenac OR diclofenac OR alclofenac OR bumadizone OR etodolac OR lonazolac OR fentiazac OR acemetacin OR difenpiramide OR oxametacine OR proglumetacin OR ketorolac OR bufexamac OR indometacin, combinations OR diclofenac, combinations OR piroxicam OR tenoxicam OR droxicam OR lornoxicam OR meloxicam OR ibuprofen OR naproxen OR ketoprofen OR fenoprofen OR fenbufen OR benoxaprofen OR suprofen OR pirprofen OR flurbiprofen OR indoprofen OR tiaprofenic acid OR oxaprozin OR ibuproxam OR dexibuprofen OR flunoxaprofen OR alminoprofen OR dexketoprofen OR ibuprofen, combinations OR ketoprofen, combinations OR mefenamic acid OR tolfenamic acid OR flufenamic acid OR meclofenamic acid OR celecoxib OR rofecoxib OR valdecoxib OR parecoxib OR etoricoxib OR lumiracoxib OR nabumetone OR niflumic acid OR azapropazone OR glucosamine OR benzydamine OR glycosaminoglycan polysulfate OR proquazone OR orgotein OR nimesulide OR feprazone OR diacerein OR morniflumate OR tenidap OR oxaceprol OR chondroitin sulfate OR feprazone

3. cohort studies OR cohort OR epidemiologic methods OR case-control studies OR (case AND control) OR risk OR incidence

4. letter [Publication Type] OR editorial [Publication Type] OR comment [Publication Type]

5. #1 AND #2 AND #3

6. #5 NOT #4

7. #6 AND limits **Entrez Date from 1990/01/01 to 2011/05/04; Humans, English**

eTable 7. Excluded studies, by reason for exclusion

Used a different reference category

Gudbjornsson B, Thorsteinsson SB, Sigvaldason H, et al. Rofecoxib, but not celecoxib, increases the risk of thromboembolic cardiovascular events in young adults-a nationwide registry-based study. *Eur J Clin Pharmacol*. 2010;66(6):619-625.

Huang WF, Hsiao FY, Tsai YW, Wen YW, Shih YT. Cardiovascular events associated with long-term use of celecoxib, rofecoxib and meloxicam in Taiwan: an observational study. *Drug Saf*. 2006;29(3):261-272.

Huang WF, Hsiao FY, Wen YW, Tsai YW. Cardiovascular events associated with the use of four nonselective NSAIDs (etodolac, nabumetone, ibuprofen, or naproxen) versus a cyclooxygenase-2 inhibitor (celecoxib): a population-based analysis in Taiwanese adults. *Clin Ther*. 2006;28(11):1827-1836.

Jick H, Kaye JA, Russmann S, Jick SS. Nonsteroidal antiinflammatory drugs and acute myocardial infarction in patients with no major risk factors. *Pharmacotherapy*. 2006;26(10):1379-1387.

Jick SS, Kaye JA, Jick H. Diclofenac and acute myocardial infarction in patients with no major risk factors. *Br J Clin Pharmacol*. 2007;64(5):662-667.

Jick SS. The risk of gastrointestinal bleed, myocardial infarction, and newly diagnosed hypertension in users of meloxicam, diclofenac, naproxen, and piroxicam. *Pharmacotherapy*. 2000;20(7):741-744.

Patel TN, Goldberg KC. Use of aspirin and ibuprofen compared with aspirin alone and the risk of myocardial infarction. *Arch Intern Med*. 2004;164(8):852-856.

Rahme E, Nedjar H. Risks and benefits of COX-2 inhibitors vs non-selective NSAIDs: does their cardiovascular risk exceed their gastrointestinal benefit? A retrospective cohort study. *Rheumatology (Oxford)*. 2007;46(3):435-438.

Rahme E, Pilote L, LeLorier J. Association between naproxen use and protection against acute myocardial infarction. *Arch Intern Med*. 2002;162(10):1111-1115.

Rahme E, Watson DJ, Kong SX, Toubouti Y, LeLorier J. Association between nonnaproxen NSAIDs, COX-2 inhibitors and hospitalization for acute myocardial infarction among the elderly: a retrospective cohort study. *Pharmacoepidemiol Drug Saf*. 2007;16(5):493-503.

Schneeweiss S, Solomon DH, Wang PS, Rassen J, Brookhart MA. Simultaneous assessment of short-term gastrointestinal benefits and cardiovascular risks of selective cyclooxygenase 2 inhibitors and nonselective nonsteroidal antiinflammatory drugs: an instrumental variable analysis. *Arthritis Rheum*. 2006;54(11):3390-3398.

Velentgas P, West W, Cannuscio CC, Watson DJ, Walker AM. Cardiovascular risk of selective cyclooxygenase-2 inhibitors and other non-aspirin non-steroidal anti-inflammatory medications. *Pharmacoepidemiol Drug Saf*. 2006;15(9):641-652.

Did not provide additional information

Fischer LM, Schlienger RG, Matter CM, Jick H, Meier CR. Current use of nonsteroidal antiinflammatory drugs and the risk of acute myocardial infarction. *Pharmacotherapy*. 2005;25(4):503-510.

Kimmel SE, Berlin JA, Reilly M, et al. The effects of nonselective non-aspirin non-steroidal anti-inflammatory medications on the risk of nonfatal myocardial infarction and their interaction with aspirin. *J Am Coll Cardiol*. 2004;43(6):985-990.

Ray WA, Stein CM, Hall K, Daugherty JR, Griffin MR. Non-steroidal anti-inflammatory drugs and risk of serious coronary heart disease: an observational cohort study. *Lancet*. 2002;359(9301):118-123.

Schlienger RG, Jick H, Meier CR. Use of nonsteroidal anti-inflammatory drugs and the risk of first-time acute myocardial infarction. *Br J Clin Pharmacol*. 2002;54(3):327-332.

Solomon DH, Schneeweiss S, Glynn RJ, et al. Relationship between selective cyclooxygenase-2 inhibitors and acute myocardial infarction in older adults. *Circulation*. 2004;109(17):2068-2073.

Watson DJ, Rhodes T, Cai B, Guess HA. Lower risk of thromboembolic cardiovascular events with naproxen among patients with rheumatoid arthritis. *Arch Intern Med*. 2002;162(10):1105-1110.

1. Forest plots for cohort and nested case-control studies, ever had an acute myocardial infarction


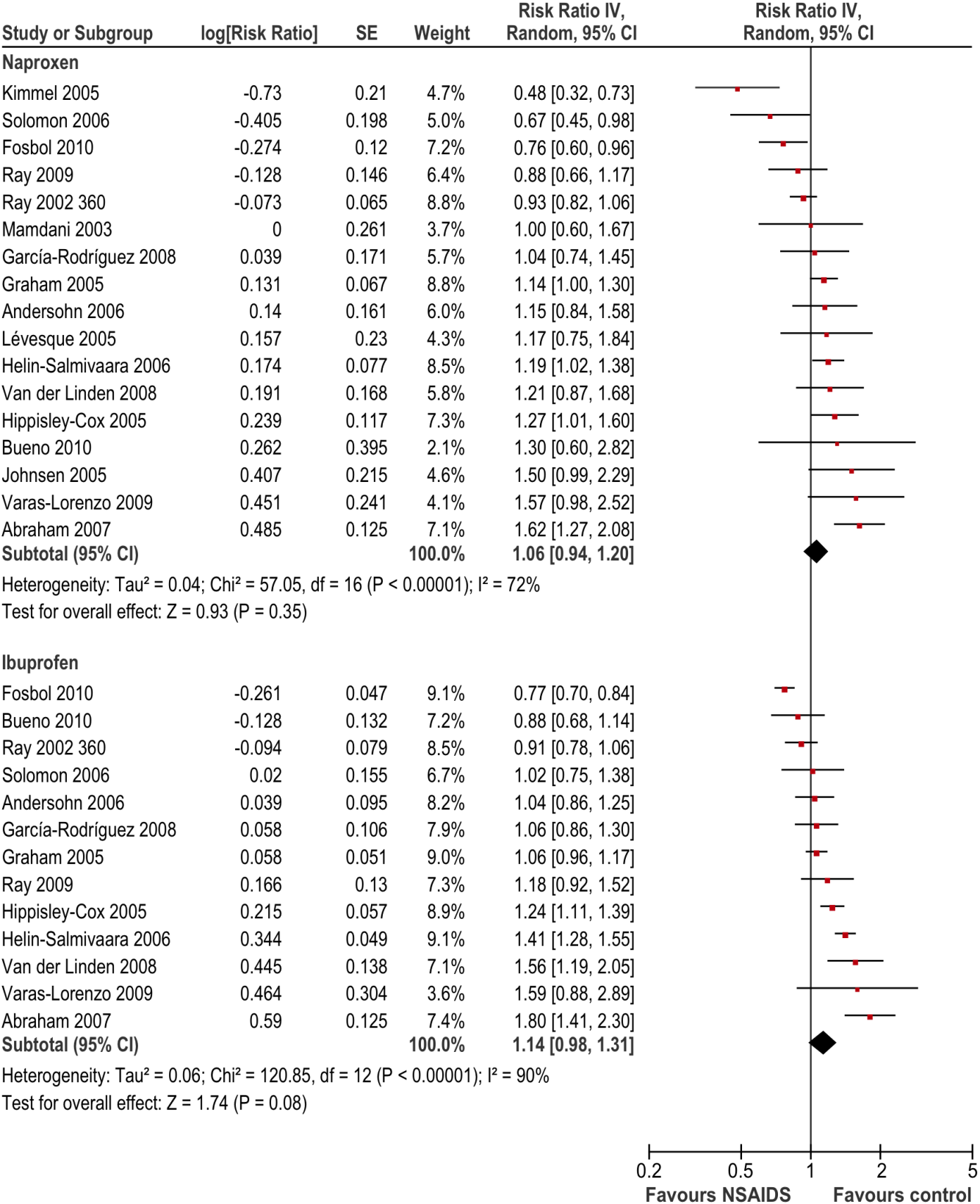


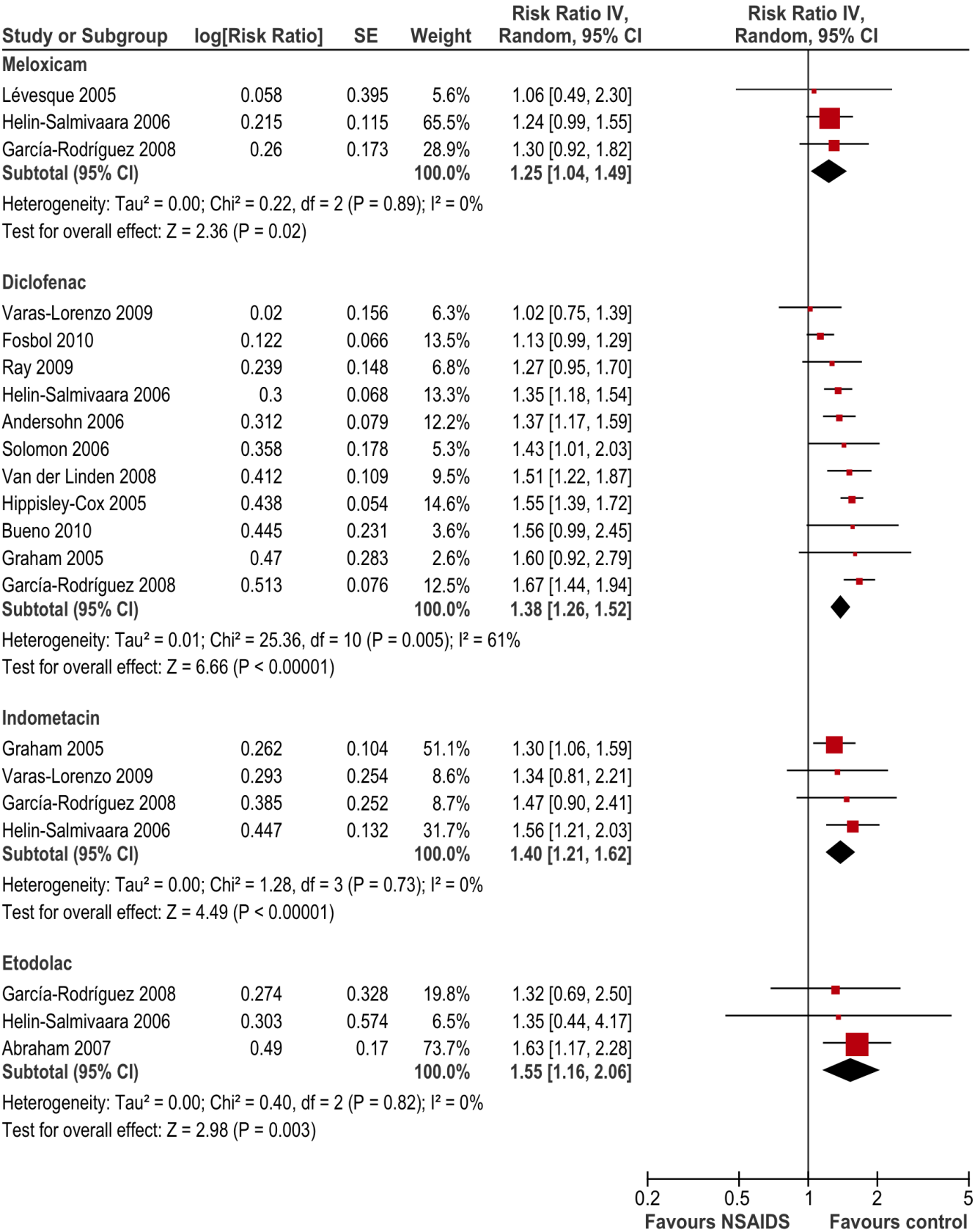


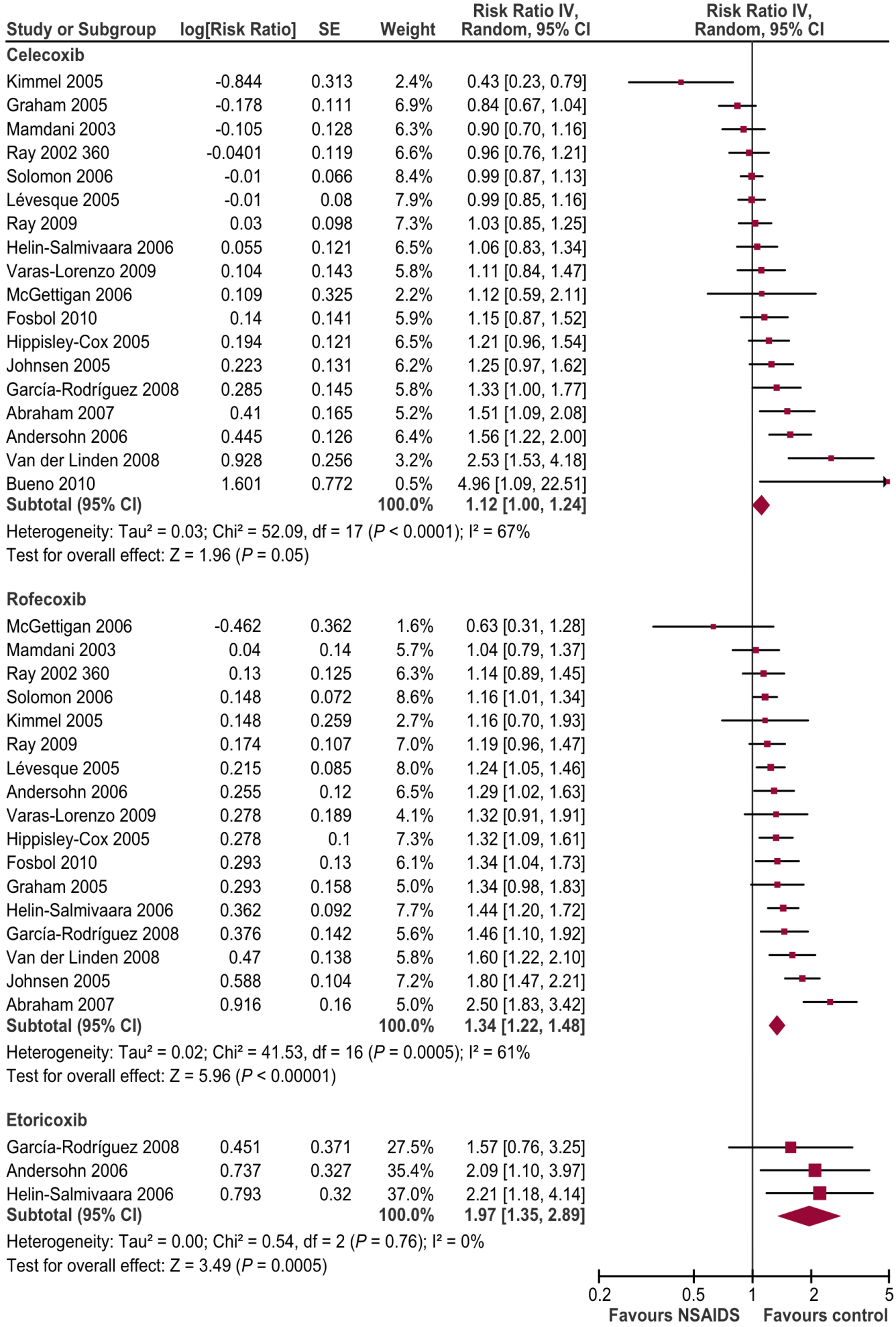


1. Funnel plot, relative risk of acute myocardial infarction for individual NSAID compared with NSAID nonuse—naproxen


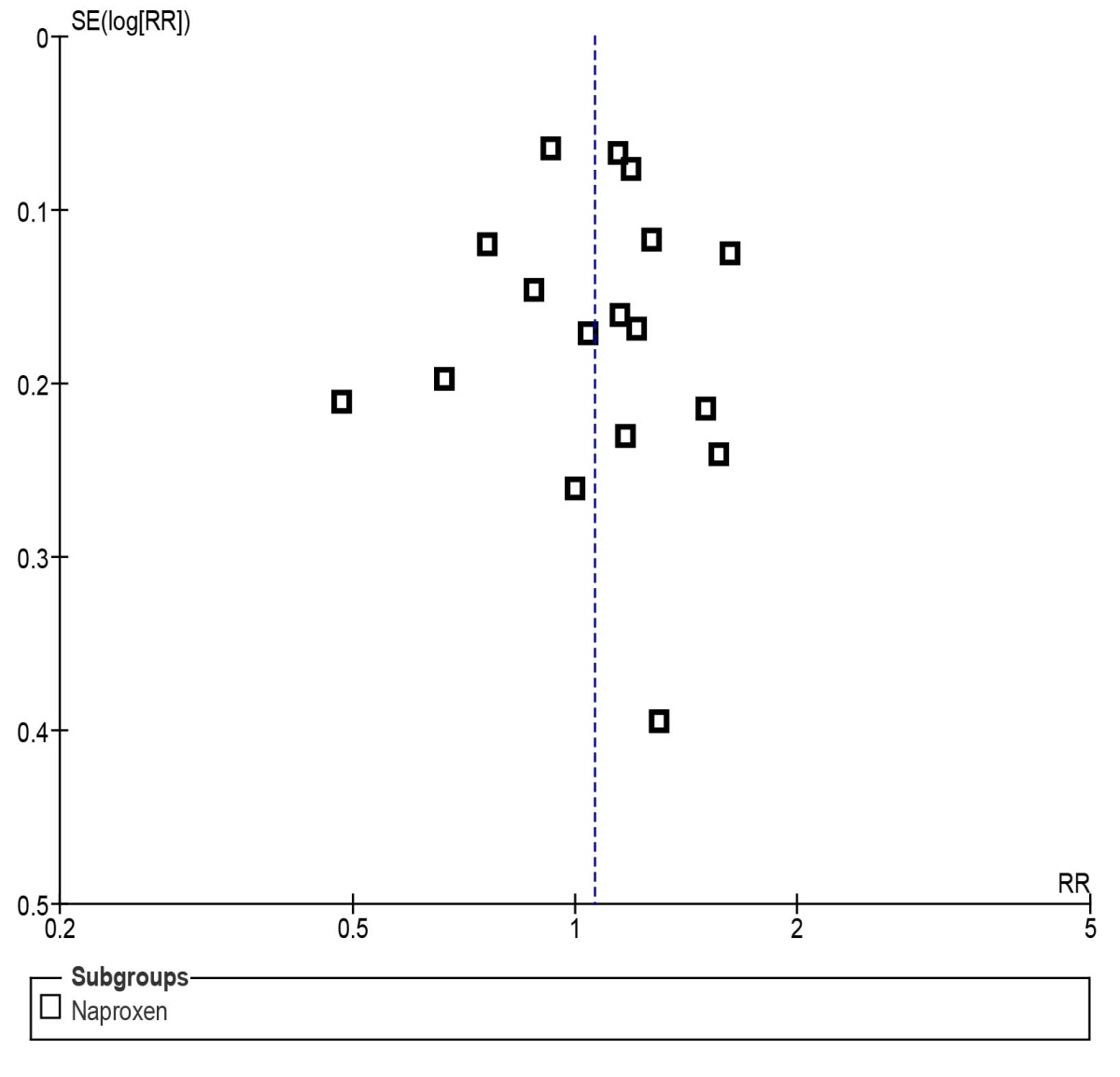


AMI = acute myocardial infarction; NSAID = nonsteroidal anti-inflammatory drug; RR = relative risk.

Note: Relative risk is plotted on the horizontal axis, and an estimate of its precision, SE(log RR), on the vertical axis.

1. Funnel plot, relative risk of acute myocardial infarction for individual NSAID compared with NSAID nonuse—ibuprofen


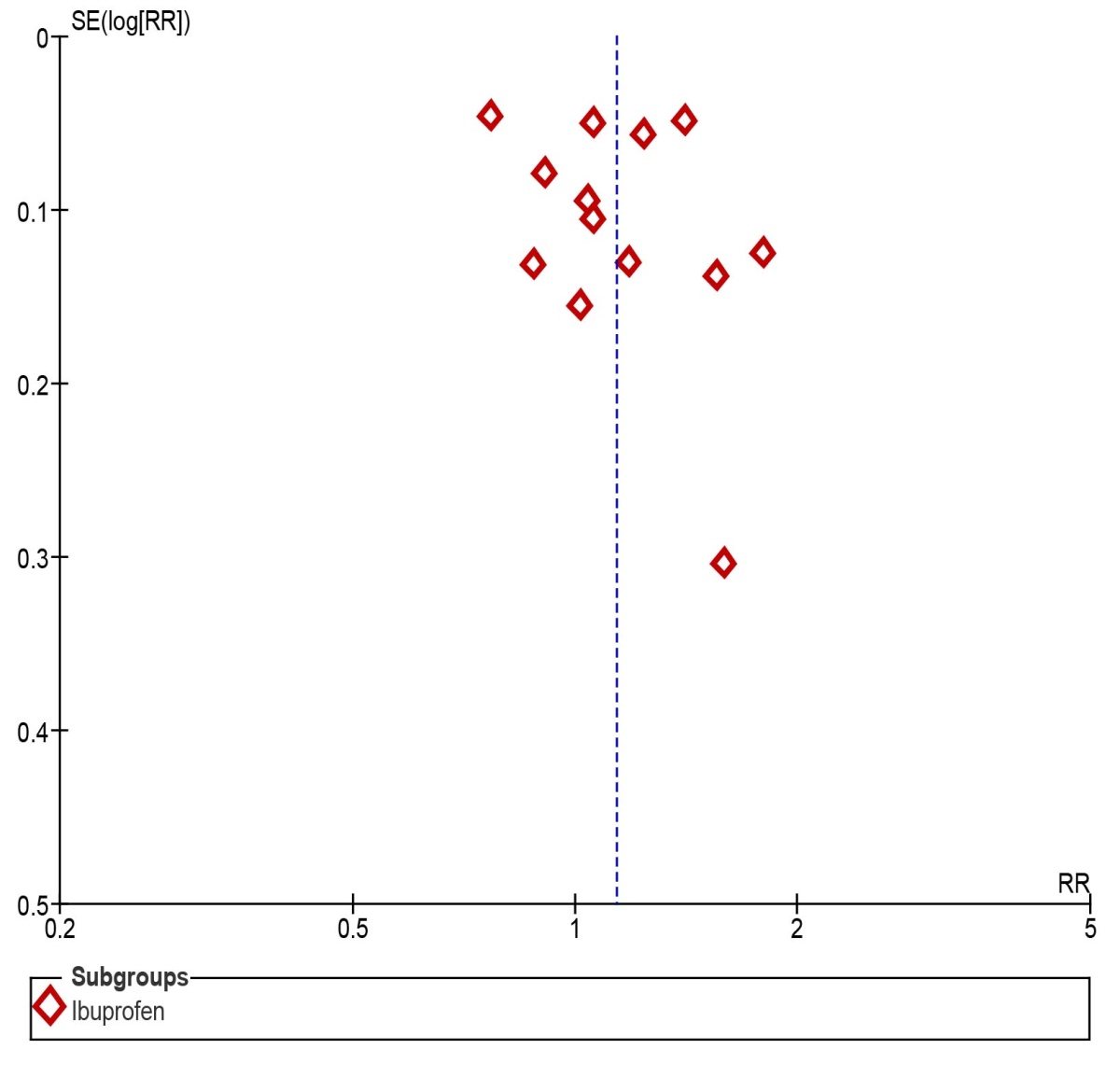


AMI = acute myocardial infarction; NSAID = nonsteroidal anti-inflammatory drug; RR = relative risk.

Note: Relative risk is plotted on the horizontal axis, and an estimate of its precision, SE(log RR), on the vertical axis.

1. Funnel plot, relative risk of acute myocardial infarction for individual NSAID compared with NSAID nonuse—meloxicam


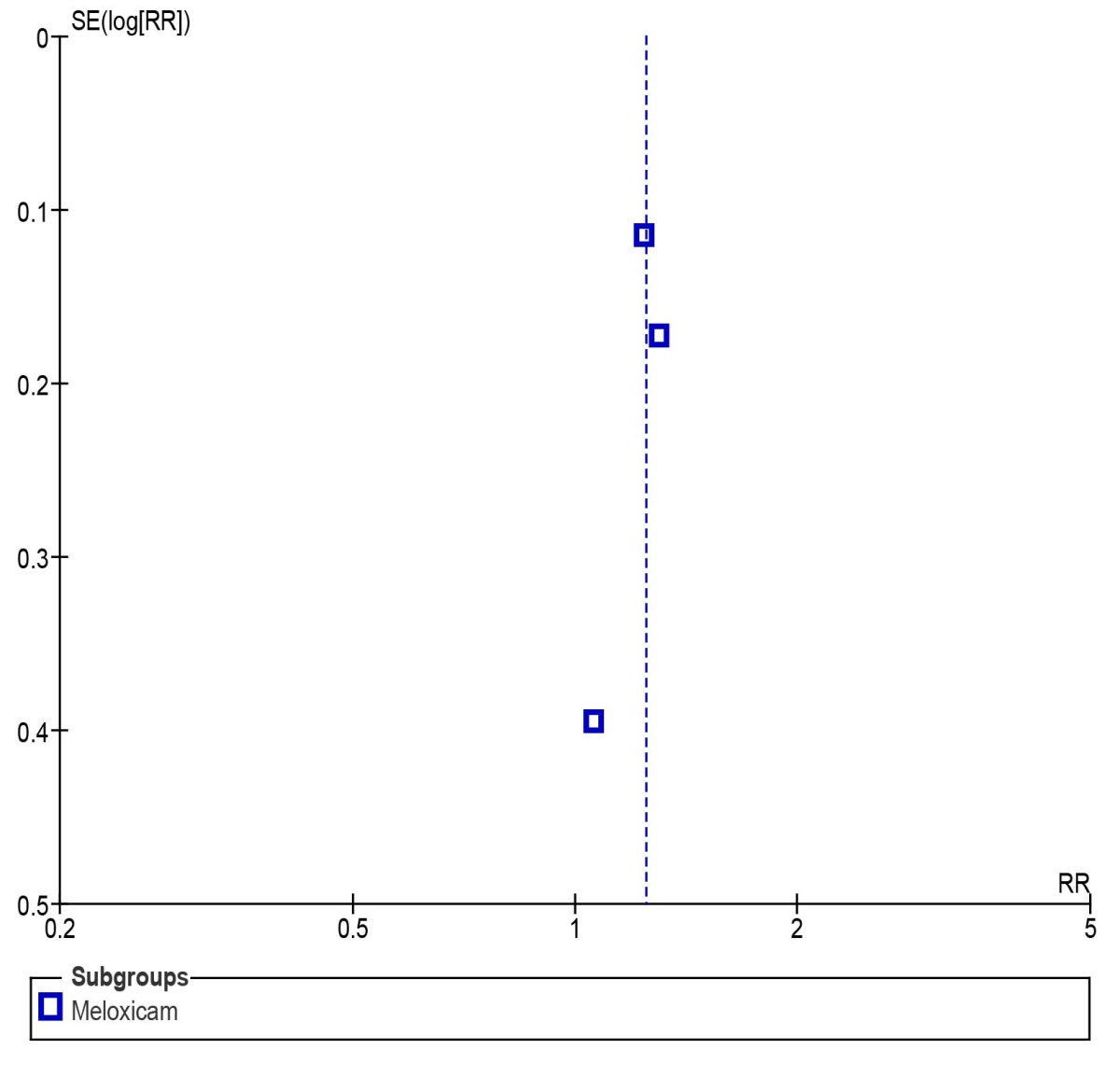


AMI = acute myocardial infarction; NSAID = nonsteroidal anti-inflammatory drug; RR = relative risk.

Note: Relative risk is plotted on the horizontal axis, and an estimate of its precision, SE(log RR), on the vertical axis.

1. Funnel plot, relative risk of acute myocardial infarction for individual NSAID compared with NSAID nonuse—diclofenac


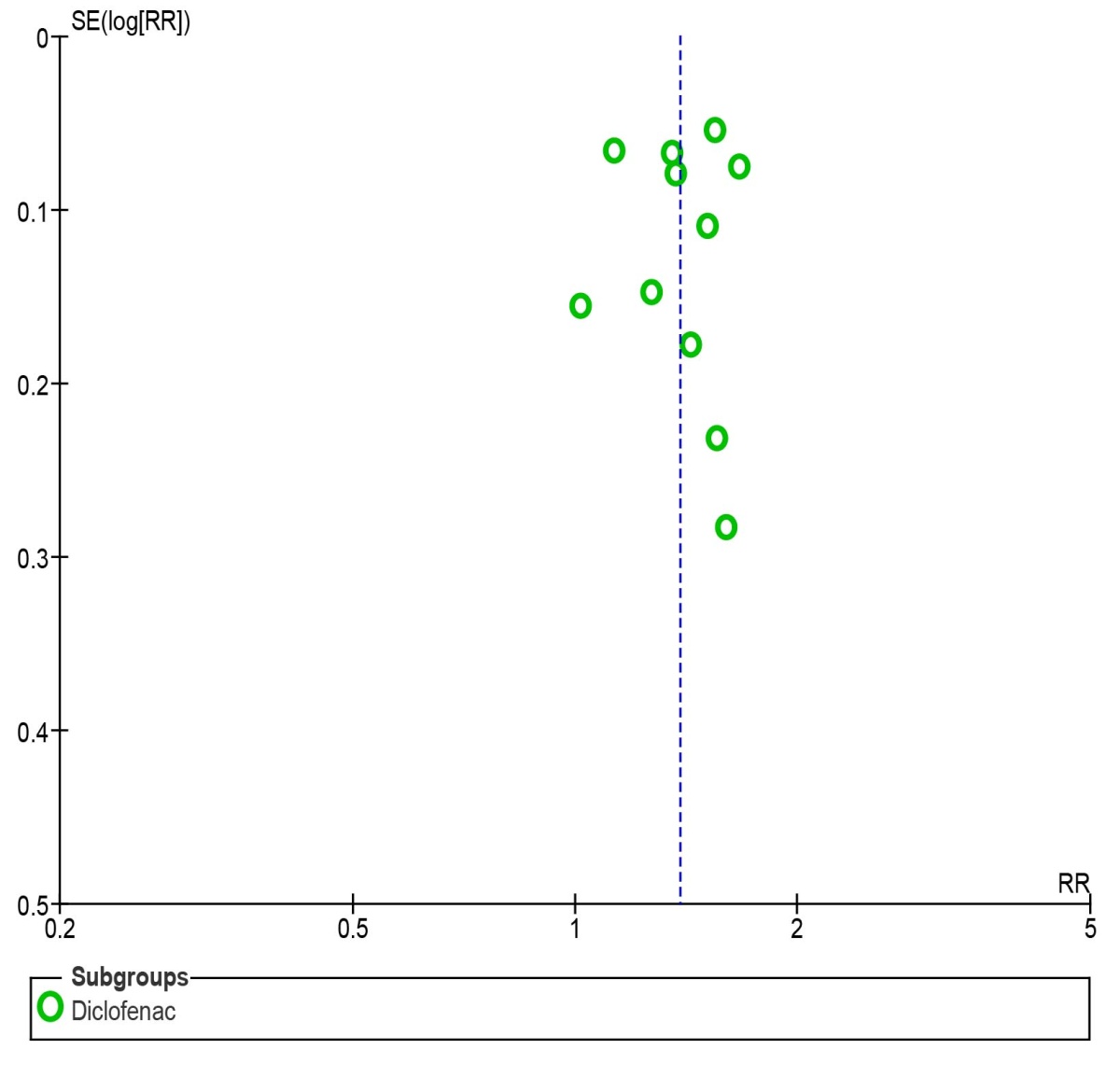


AMI = acute myocardial infarction; NSAID = nonsteroidal anti-inflammatory drug; RR = relative risk.

Note: Relative risk is plotted on the horizontal axis, and an estimate of its precision, SE(log RR), on the vertical axis.

1. Funnel plot, relative risk of acute myocardial infarction for individual NSAID compared with NSAID nonuse—indometacin


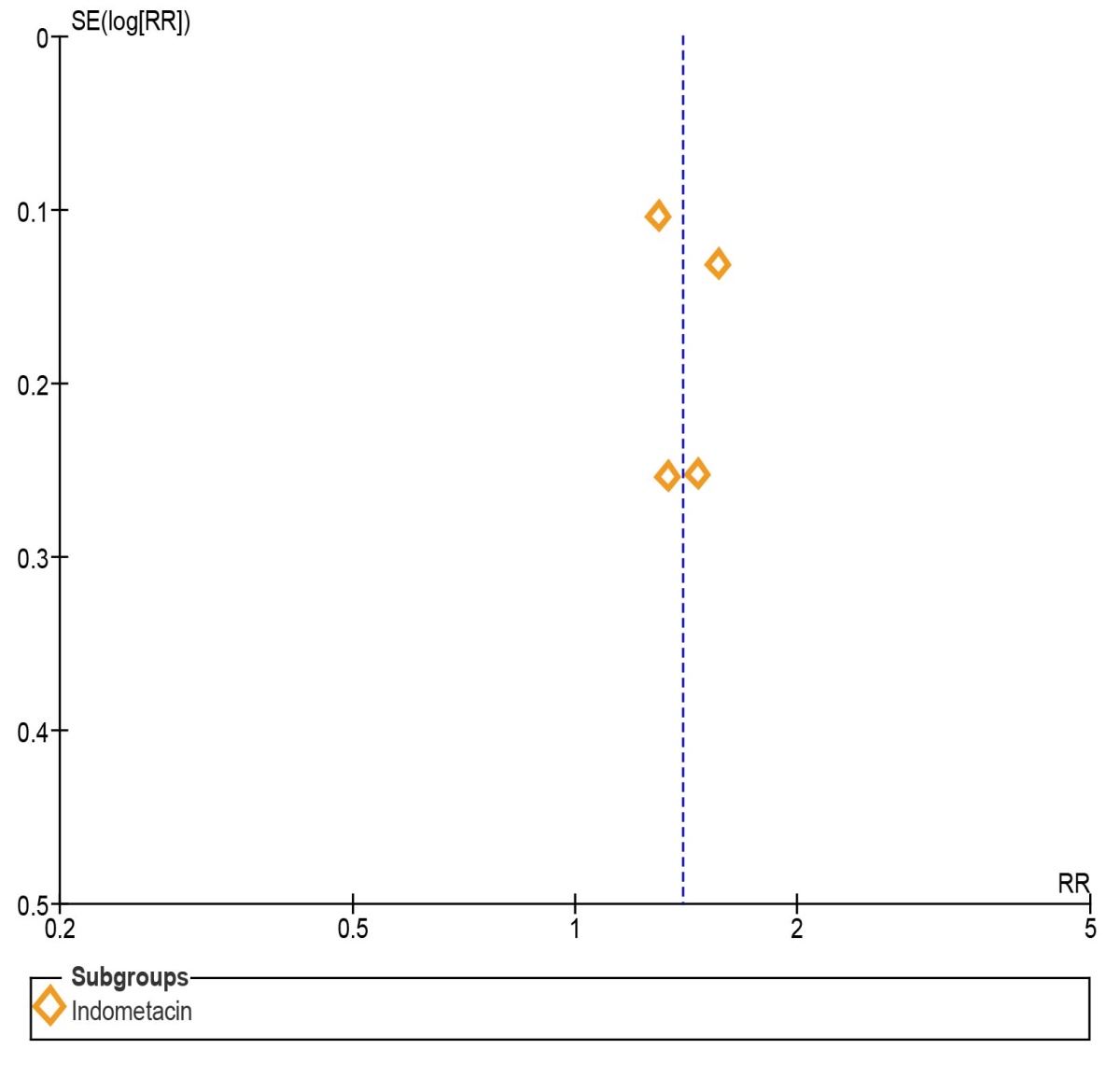


AMI = acute myocardial infarction; NSAID = nonsteroidal anti-inflammatory drug; RR = relative risk.

Note: Relative risk is plotted on the horizontal axis, and an estimate of its precision, SE(log RR), on the vertical axis.

1. Funnel plot, relative risk of acute myocardial infarction for individual NSAID compared with NSAID nonuse—etodolac


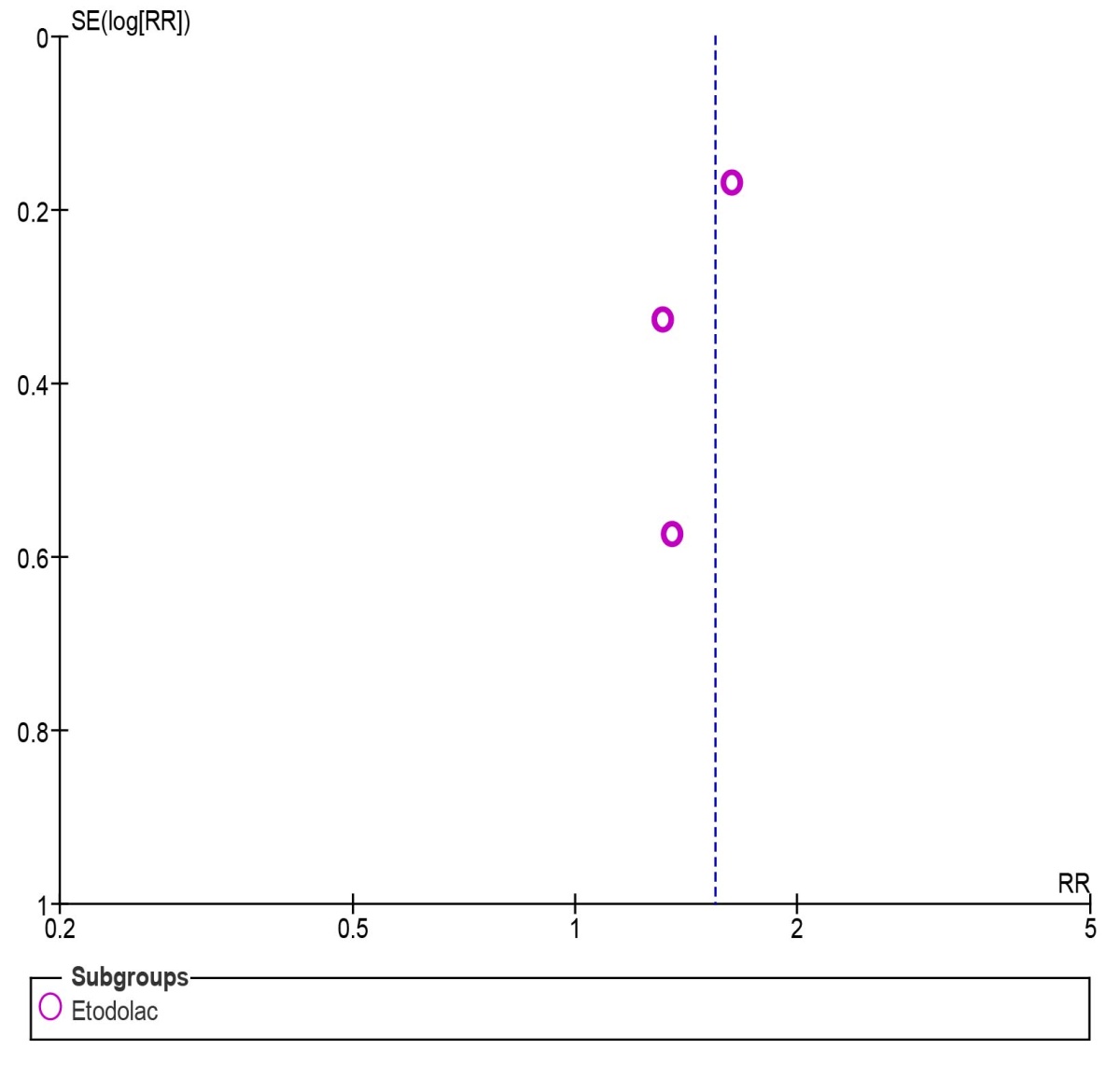


AMI = acute myocardial infarction; NSAID = nonsteroidal anti-inflammatory drug; RR = relative risk.

Note: Relative risk is plotted on the horizontal axis, and an estimate of its precision, SE(log RR), on the vertical axis.

1. Funnel plot, relative risk of acute myocardial infarction for individual NSAID compared with NSAID nonuse—celecoxib


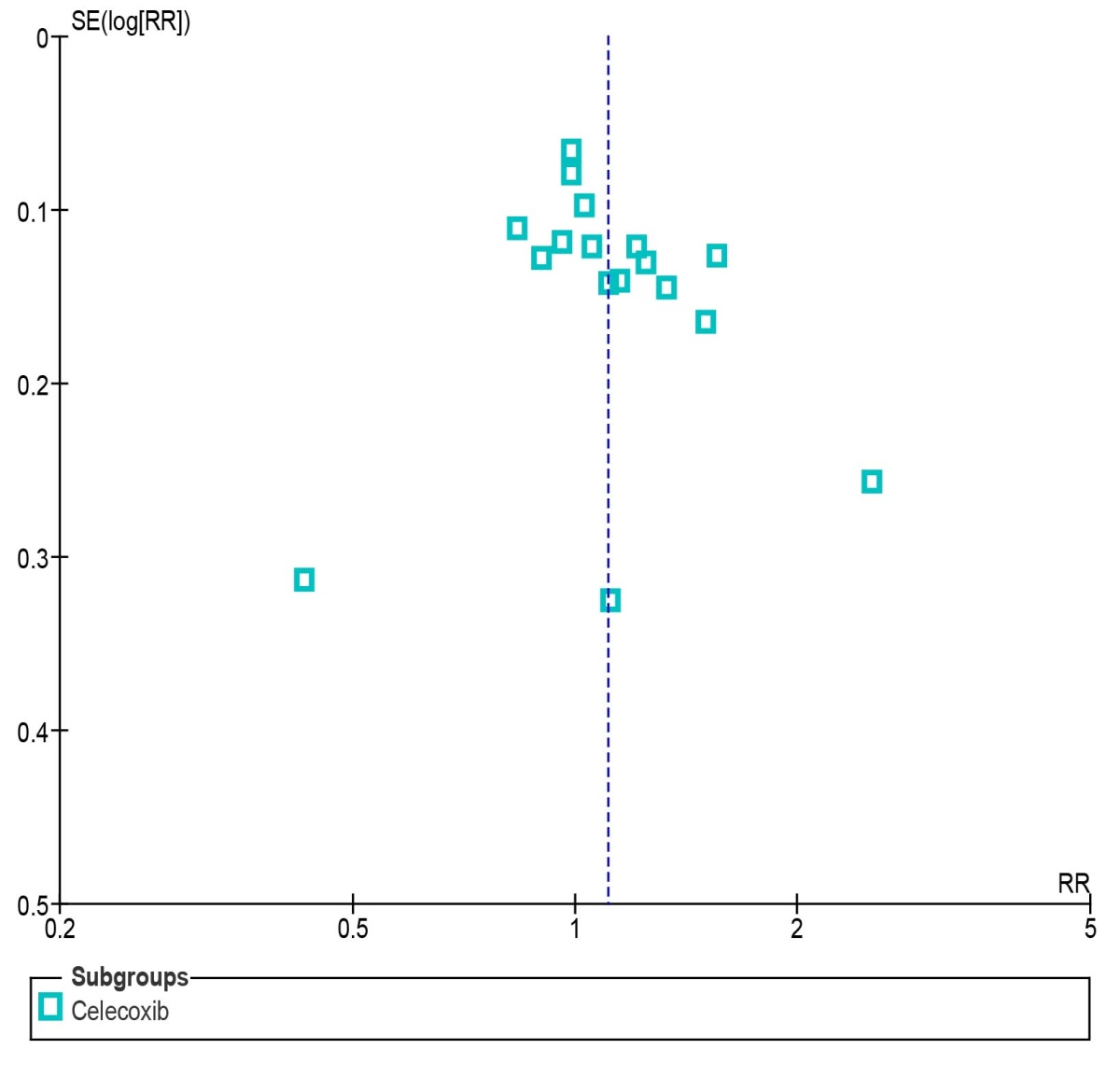


AMI = acute myocardial infarction; NSAID = nonsteroidal anti-inflammatory drug; RR = relative risk.

Note: Relative risk is plotted on the horizontal axis, and an estimate of its precision, SE(log RR), on the vertical axis.

1. Funnel plot, relative risk of acute myocardial infarction for individual NSAID compared with NSAID nonuse—rofecoxib


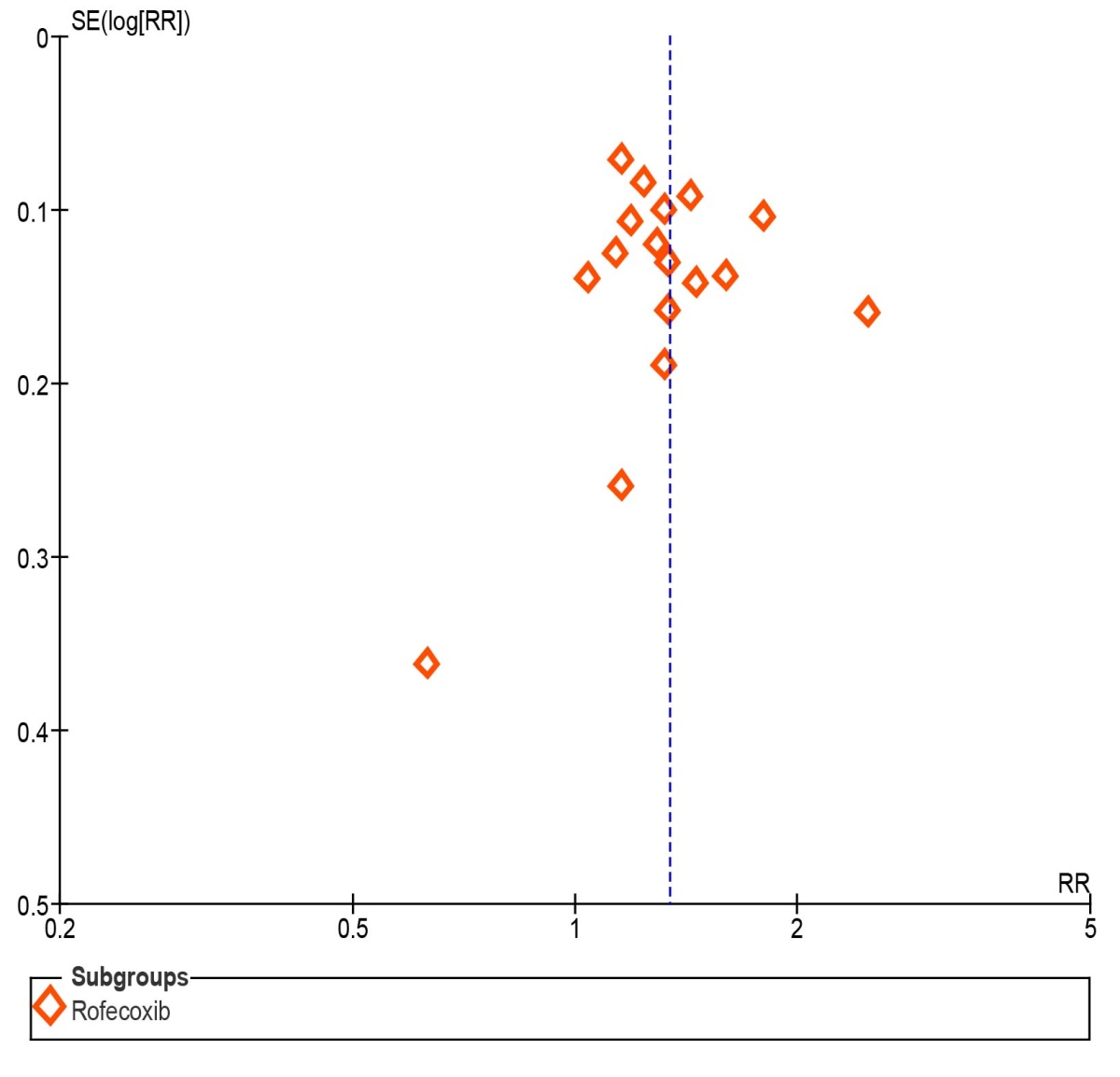


AMI = acute myocardial infarction; NSAID = nonsteroidal anti-inflammatory drug; RR = relative risk.

Note: Relative risk is plotted on the horizontal axis, and an estimate of its precision, SE(log RR), on the vertical axis.

1. Funnel plot, relative risk of acute myocardial infarction for individual NSAID compared with NSAID nonuse—etoricoxib


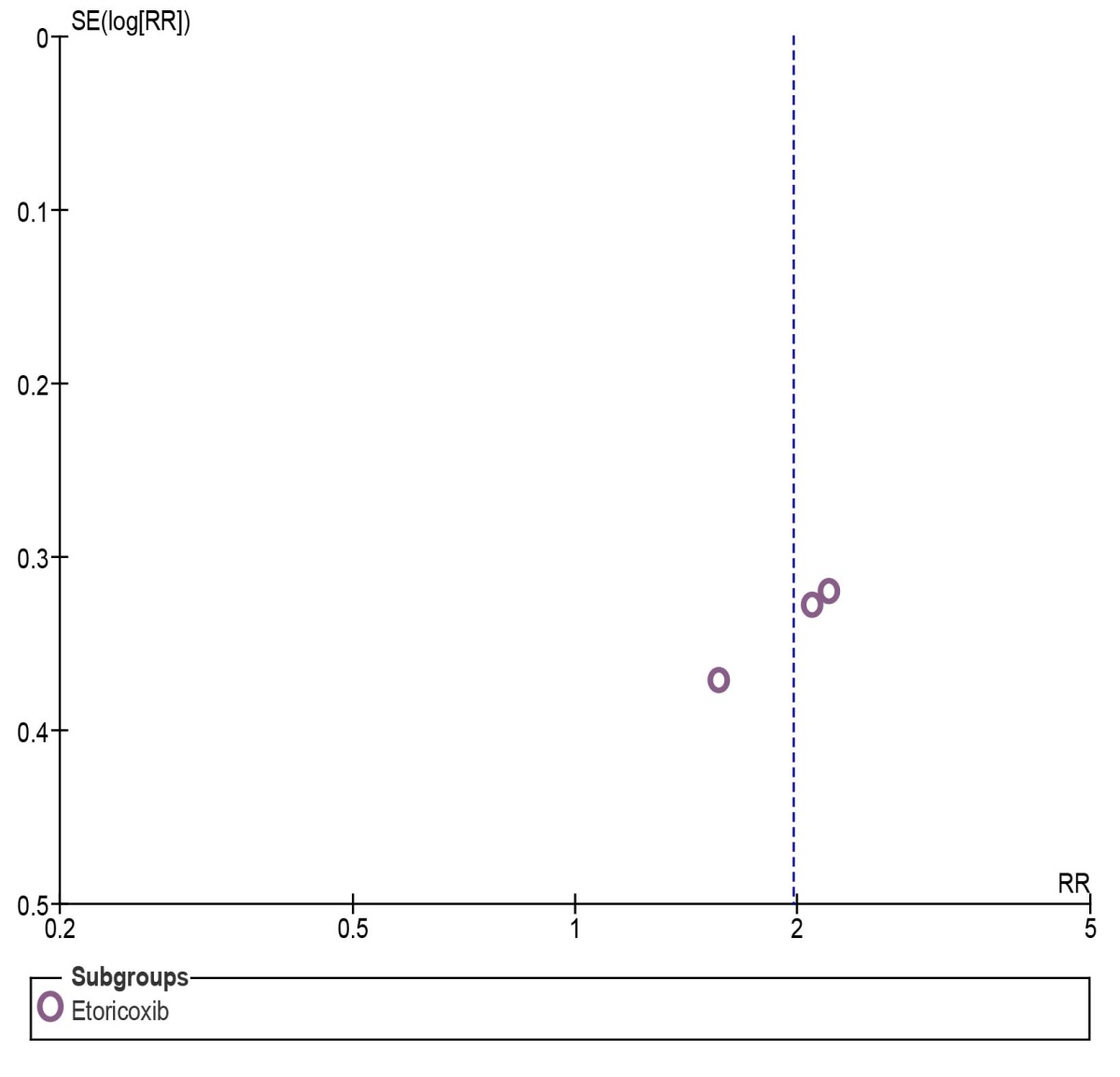


AMI = acute myocardial infarction; NSAID = nonsteroidal anti-inflammatory drug; RR = relative risk.

Note: Relative risk is plotted on the horizontal axis, and an estimate of its precision, SE(log RR), on the vertical axis.

# References for Supplemental Material

1. Abraham NS, El-Serag HB, Hartman C, Richardson P, Deswal A. Cyclooxygenase-2 selectivity of non-steroidal anti-inflammatory drugs and the risk of myocardial infarction and cerebrovascular accident. Aliment Pharmacol Ther 2007;25:913-24.

2. Solomon DH, Glynn RJ, Levin R, Avorn J. Nonsteroidal anti-inflammatory drug use and acute myocardial infarction. Arch Intern Med 2002;162:1099-104.

3. Solomon DH, Schneeweiss S, Glynn RJ, Kiyota Y, Levin R, Mogun H, et al. Relationship between selective cyclooxygenase-2 inhibitors and acute myocardial infarction in older adults. Circulation 2004;109:2068-73.

4. Solomon DH, Avorn J, Stürmer T, Glynn RJ, Mogun H, Schneeweiss S. Cardiovascular outcomes in new users of coxibs and nonsteroidal antiinflammatory drugs: high-risk subgroups and time course of risk. Arthritis Rheum 2006;54:1378-89.

5. Ray WA, Stein CM, Hall K, Daugherty JR, Griffin MR. Non-steroidal anti-inflammatory drugs and risk of serious coronary heart disease: an observational cohort study. Lancet 2002;359:118-23.

6. Ray WA, Stein CM, Daugherty JR, Hall K, Arbogast PG, Griffin MR. COX-2 selective non-steroidal anti-inflammatory drugs and risk of serious coronary heart disease. Lancet 2002;360:1071-3.

7. Graham DJ, Campen D, Hui R, Spence M, Cheetham C, Levy G, et al. Risk of acute myocardial infarction and sudden cardiac death in patients treated with cyclo-oxygenase 2 selective and non-selective non-steroidal anti-inflammatory drugs: nested case-control study. Lancet 2005;365:475-81.

8. Kimmel SE, Berlin JA, Reilly M, Jaskowiak J, Kishel L, Chittams J, et al. The effects of nonselective non-aspirin non-steroidal anti-inflammatory medications on the risk of nonfatal myocardial infarction and their interaction with aspirin. J Am Coll Cardiol 2004;43:985-90.

9. Kimmel SE, Berlin JA, Reilly M, Jaskowiak J, Kishel L, Chittams J, et al. Patients exposed to rofecoxib and celecoxib have different odds of nonfatal myocardial infarction. Ann Intern Med 2005;142:157-64.

10. Varas-Lorenzo C, Castellsague J, Stang MR, Perez-Gutthann S, Aguado J, Rodriguez LA. The use of selective cyclooxygenase-2 inhibitors and the risk of acute myocardial infarction in Saskatchewan, Canada. Pharmacoepidemiol Drug Saf 2009;18:1016-25.

11. Lévesque LE, Brophy JM, Zhang B. The risk for myocardial infarction with cyclooxygenase-2 inhibitors: a population study of elderly adults. Ann Intern Med 2005;142:481-9.

12. Lévesque LE, Brophy JM, Zhang B. Time variations in the risk of myocardial infarction among elderly users of COX-2 inhibitors. CMAJ 2006;174:1563-9.

13. Brophy JM, Lévesque LE, Zhang B. The coronary risk of cyclo-oxygenase-2 inhibitors in patients with a previous myocardial infarction. Heart 2007;93:189-94.

14. Mamdani M, Rochon P, Juurlink DN, Anderson GM, Kopp A, Naglie G, et al. Effect of selective cyclooxygenase 2 inhibitors and naproxen on short-term risk of acute myocardial infarction in the elderly. Arch Intern Med 2003;163:481-6.

15. McGettigan P, Han P, Henry D. Cyclooxygenase-2 inhibitors and coronary occlusion--exploring dose-response relationships. Br J Clin Pharmacol 2006;62:358-65.

16. Watson DJ, Rhodes T, Cai B, Guess HA. Lower risk of thromboembolic cardiovascular events with naproxen among patients with rheumatoid arthritis. Arch Intern Med 2002;162:1105-10.

17. Schlienger RG, Jick H, Meier CR. Use of nonsteroidal anti-inflammatory drugs and the risk of first-time acute myocardial infarction. Br J Clin Pharmacol 2002;54:327-32.

18. Fischer LM, Schlienger RG, Matter CM, Jick H, Meier CR. Current use of nonsteroidal antiinflammatory drugs and the risk of acute myocardial infarction. Pharmacotherapy 2005;25:503-10.

19. García Rodríguez LA, Varas-Lorenzo C, Maguire A, González-Pérez A. Nonsteroidal antiinflammatory drugs and the risk of myocardial infarction in the general population. Circulation 2004;109:3000-6.

20. García Rodríguez LA, González-Pérez A. Long-term use of non-steroidal anti-inflammatory drugs and the risk of myocardial infarction in the general population. BMC Med 2005;3:17.

21. Andersohn F, Suissa S, Garbe E. Use of first- and second-generation cyclooxygenase-2-selective nonsteroidal antiinflammatory drugs and risk of acute myocardial infarction. Circulation 2006;113:1950-7.

22. García Rodríguez LA, Tacconelli S, Patrignani P. Role of dose potency in the prediction of risk of myocardial infarction associated with nonsteroidal anti-inflammatory drugs in the general population. J Am Coll Cardiol 2008;52:1628-36.

23. Hippisley-Cox J, Coupland C. Risk of myocardial infarction in patients taking cyclo-oxygenase-2 inhibitors or conventional non-steroidal anti-inflammatory drugs: population based nested case-control analysis. BMJ 2005;330:1366.

24. Fosbøl EL, Folke F, Jacobsen S, Rasmussen JN, Sørensen R, Schramm TK, et al. Cause-specific cardiovascular risk associated with nonsteroidal antiinflammatory drugs among healthy individuals. Circ Cardiovasc Qual Outcomes 2010;3:395-405.

25. Schjerning Olsen AM, Fosbøl EL, Lindhardsen J, Folke F, Charlot M, Selmer C, et al. Duration of treatment with nonsteroidal anti-inflammatory drugs and impact on risk of death and recurrent myocardial infarction in patients with prior myocardial infarction: a nationwide cohort study. Circulation 2011;123:2226-35.

26. Gislason GH, Jacobsen S, Rasmussen JN, Rasmussen S, Buch P, Friberg J, et al. Risk of death or reinfarction associated with the use of selective cyclooxygenase-2 inhibitors and nonselective nonsteroidal antiinflammatory drugs after acute myocardial infarction. Circulation 2006;113:2906-13.

27. Helin-Salmivaara A, Virtanen A, Vesalainen R, Grönroos JM, Klaukka T, Idänpään-Heikkilä JE, et al. NSAID use and the risk of hospitalization for first myocardial infarction in the general population: a nationwide case-control study from Finland. Eur Heart J 2006;27:1657-63.

28. Van der Linden MW, Van der Bij S, Welsing P, Kuipers EJ, Herings RM. The balance between severe cardiovascular and gastrointestinal events among users of selective and non-selective non steroidal anti-inflammatory drugs. Ann Rheum Dis 2008;68:668-73.

29. Johnsen SP, Larsson H, Tarone RE, McLaughlin JK, Nørgård B, Friis S, et al. Risk of hospitalization for myocardial infarction among users of rofecoxib, celecoxib, and other NSAIDs: a population-based case-control study. Arch Intern Med 2005;165:978-84.

30. Bueno H, Bardají A, Patrignani P, Martín-Merino E, García-Rodríguez LA. Use of non-steroidal antiinflammatory drugs and type-specific risk of acute coronary syndrome. Am J Cardiol 2010;105:1102-6.

31. Ray WA, Varas-Lorenzo C, Chung CP, Castellsague J, Murray KT, Stein CM, et al. Cardiovascular risks of nonsteroidal antiinflammatory drugs in patients after hospitalization for serious coronary heart disease. Circ Cardiovasc Qual Outcomes 2009;2:155-63.

32. Moher D, Liberati A, Tetzlaff J, Altman DG. Preferred reporting items for systematic reviews and meta-analyses: the PRISMA statement. PLoS Med 2009;6:e1000097.
